# A Review of the Current Development State of Non-Terrestrial NB-IoT Systems

**DOI:** 10.3390/s26165274

**Published:** 2026-08-20

**Authors:** Vitalii Beschastnyi, Uliana Morozova, Darya Ostrikova, Yuliya Gaidamaka, Konstantin Samouylov

**Affiliations:** Department of Probability Theory and Cybersecurity, Peoples’ Friendship University of Russia (RUDN University), 117198 Moscow, Russia; morozova-uk@rudn.ru (U.M.); ostrikova-dyu@rudn.ru (D.O.); gaydamaka-yuv@rudn.ru (Y.G.); samuylov-ke@rudn.ru (K.S.)

**Keywords:** 5G, NTN, NB-IoT, orbiting systems, satellites, deployment, lessons

## Abstract

The Internet of Things (IoT) market is currently undergoing a period of unprecedented, rapid evolution, leading to the enabling of novel and diverse applications spanning both the civilian and industrial sectors. A significant proportion of these emerging use cases, particularly those in domains such as maritime communications and forestry management, require service continuity and connectivity within geographically remote regions, where conventional terrestrial infrastructure is often absent or economically unfeasible. To bridge this coverage gap and achieve truly ubiquitous connectivity, the recent 3GPP initiative to extend 5G services into Non-Terrestrial Segments (NTNs) holds substantial promise. This expansion is crucial for ensuring that massive Machine-Type Communication (mMTC) services can be reliably provisioned globally. This paper aims to detail the progress in standardization and academic activities towards the design and deployment of NTN-based Narrowband IoT (NB-IoT) systems, which are the leading NTN mMTC enabler in the 3GPP portfolio. We will specify the challenges faced by these systems and outline the solutions proposed thus far. We conclude the paper with a discussion on already operational systems and lessons learned from their deployment and operation.

## 1. Introduction

The Narrowband Internet of Things (NB-IoT) has emerged as the foundational standard for a broad category of IoT deployments, characterized by relaxed constraints on both communication latency and data throughput [1,2]. Standardized initially within 3GPP Release 13 and subsequently refined, NB-IoT operates over highly narrow channel bandwidths—specifically, 200 kHz (comprising 180 kHz of usable bandwidth framed by two 10 kHz guard bands), which is perfectly aligned with the spectral allocation of a single legacy GSM channel [1]. This design facilitates the use of highly simplified End Devices (EDs), achieving a complexity reduction of 80% to 90% compared with LTE Cat-1 devices [3,4].

When implemented in a Terrestrial Network (TN) configuration, NB-IoT leverages sophisticated coverage enhancement and power-saving mechanisms to deliver extensive coverage of up to 30 km [5]. Crucially, it supports an unprecedented density of over 50,000 networked EDs per cell (assuming a low traffic profile of only a few messages per 24 h) and guarantees exceptional battery longevity, often exceeding 10 years [5]. Collectively, these technical attributes position NB-IoT as the ideal technology for large-scale low-power wide-area (LPWA) IoT applications [5].

Despite the sustained deployment of wireless communication systems, approximately 85% of the Earth’s surface remains disconnected and is beyond the operational range of conventional terrestrial communication systems. Concurrently, there is an unprecedented surge in demand for massive Machine-Type Communication (mMTC) services across critical industrial sectors, including forestry, maritime logistics, and global container tracking, many of which inherently operate within these geographically remote or isolated areas.

**Figure 1:**
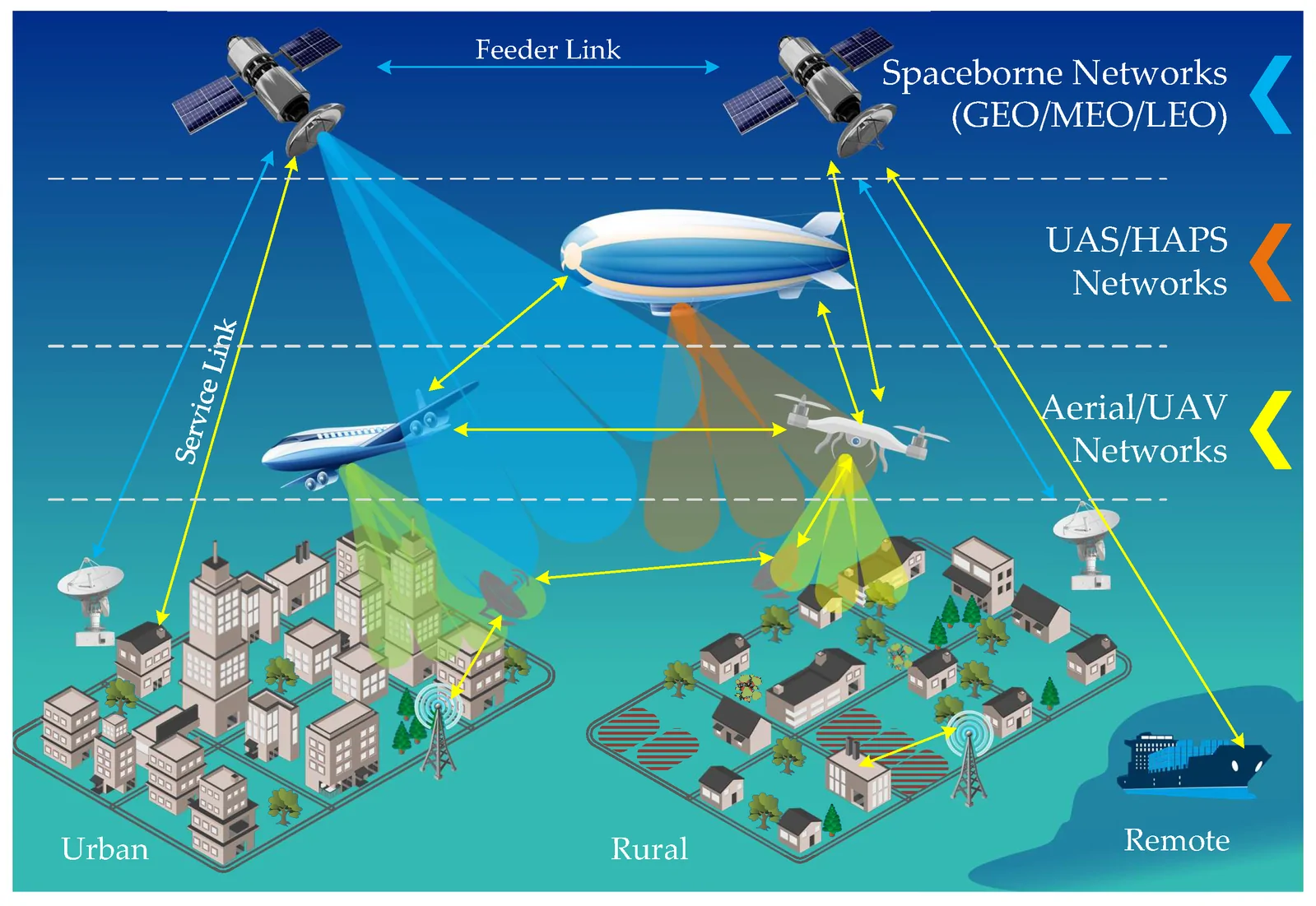
An ecosystem of NTN NB-IoT services and delivery layers [6].

To effectively address this global coverage deficit and mitigate the need for economically prohibitive ground-based infrastructure, the integration of Non-Terrestrial Networks (NTNs) has been formally proposed and initiated as a core component of the broader 5G framework. NTNs utilize a diverse range of aerial and orbital platforms, specifically High-Altitude Platform Stations (HAPSs) and various satellite constellations, including low-earth orbit (LEO), medium-earth orbit (MEO), and Geostationary Orbit (GEO) satellites. This infrastructure offers significant potential for extending mMTC service coverage to previously inaccessible regions, as shown in Figure 1. Within this strategic shift toward global connectivity, the deployment of NB-IoT technology over NTNs represents a particularly promising avenue for realizing ubiquitous massive Machine-Type Communications (mMTCs).

The initial developmental push to integrate the Non-Terrestrial Network (NTN) segment into cellular systems originated with 3GPP Release 14. This first wave of standardization primarily focused on a foundational assessment of technical feasibility, driven chiefly by the economic imperative to extend network coverage into geographically sparse or less densely populated regions where the deployment of conventional terrestrial infrastructure (TN) is financially unsustainable for network operators. Following this initial assessment, the emphasis in 3GPP Release 16 shifted markedly towards enabling enhanced Mobile Broadband (eMBB) service support within the NTN framework. The formal adoption and realization of massive Machine-Type Communications (mMTCs) support via NTNs was officially materialized in Release 17. Although all NTN deployments face general integration challenges, the specific characteristics of mMTC introduce a unique set of complexities. These complications stem from the inherent design limitations of mMTC devices, which are engineered for maximum spectral efficiency and a prolonged operational life. Such limitations include severely constrained hardware capabilities, reliance on power-saving modes (PSMs), and the use of specialized transmission techniques. Recognizing these constraints, the 3GPP has progressively addressed these integration issues in subsequent standardization cycles. Specifically, 3GPP Releases 18 and 19 introduced significant refinements focusing on sophisticated mechanisms for mobility handling, robust coverage recovery, and energy-efficient scheduling, all of which are tailored to the demanding environment of satellite-based mMTC scenarios.

Despite the significant progress made to date, there are still a plethora of challenges that need to be addressed. Specifically, NTN NB-IoT still utilizes conventional random access schemes, including 4-step grant-based and modified 2-step grant-free, which are not optimized for NTN operations [7,8,9]. A critical obstacle hindering the widespread deployment of mMTC NTN is the exceptionally high capital expenditure (CapEx) required to establish comprehensive satellite constellations. Achieving reliable and ubiquitous global coverage often necessitates the launch of tens or even hundreds of satellites across diverse orbital layers (e.g., LEO and MEO) [10]. For instance, the European Union’s ambitious IRIS^2^ program aims to ensure resilient coverage across the European continent by deploying a constellation projected to contain approximately 300 satellites in a combined LEO and MEO architecture [11,12]. Consequently, in the face of such enormous investment costs, many service providers opt for gradual, phased satellite deployment strategies. However, this incremental approach invariably leads to the persistence of intermittent connectivity in specific geographic areas of interest rather than immediate, seamless coverage.

The main contributions of our study are as follows:

- Comprehensive Review of NTN NB-IoT Progress: The paper provides a detailed overview of the recent developments in both standardization (3GPP) and academia regarding the provision of NTN connectivity for NB-IoT mMTC systems.
- Analysis of Satellite Infrastructure and Deployment: It offers an in-depth classification and technical description of orbiting systems, including LEO, MEO, and GEO satellites, and evaluates their specific roles, advantages, and integration challenges within a multi-orbit 5G ecosystem.
- Synthesis of Lessons Learned from Operational Systems: This study concludes by analyzing already deployed systems and field trials, highlighting critical lessons regarding hardware validation, service performance including latency, throughput and age-of-information (AoI), software-defined infrastructure flexibility, and the necessary trade-offs between coverage, capacity, and latency.

One specific topic that is not addressed in this study is security. In our opinion, this topic requires a paper of its own because of the large body of research literature. Briefly, there are security challenges related to the overall NB-IoT design and also to the NTN segment as well. Because they share the same infrastructure owned by LTE systems, the former are also inherent to NTN NB-IoT; see [13,14] as a starting point. NTN challenges are generally shared by any 3GPP-based NTN systems including NR and NB-IoT; see [15,16] for initial reading.

Observe that [17,18] also provide overviews of NTN NB-IoT systems. Unlike [17], which provides a broad but high-level overview of general NTN enablers, and [18], which focuses on architectural and standardization frameworks, our survey focuses on the specific physical and MAC layer challenges of NB-IoT. We provide quantitative hardware parameters (e.g., power budgets and radiation hardening), detailed performance metrics (e.g., packet delivery ratio, latency, and AoI), and concrete lessons learned from commercial deployments (e.g., Airtel’s smart meters and Iridium’s direct-to-device service). Furthermore, our paper offers rigorous treatment of satellite infrastructure, including mass-based classifications and orbital dynamics, making it the definitive reference for engineers and researchers working directly on satellite-based mMTC systems.

The remainder of this paper is organized as follows. In Section 2, we cover aspects related to the use of orbiting systems for NTN. In Section 3, we cover services and outline challenges related to NB-IoT deployment and performance. The architecture and progress of the 3GPP standardization are outlined in Section 4. Academic contributions to the design and development of NTN NB-IoT systems addressing the challenges from Section 3 are presented in Section 5. The already deployed systems and lessons learned from their operation are discussed in Section 6. Finally, the conclusions are outlined in Section 7.

## 2. Orbiting Systems, Constellations, and Hardware

This section covers the critical preliminaries for the review, including an overview of the satellite infrastructure that enables NTN connectivity. We begin by establishing a unified classification of satellites based on their mass, as this factor directly dictates the launch costs, mission complexity, and development timelines. Then, we proceed to detail the specific orbital regimes—LEO, MEO, and GEO—explaining how each altitude offers different trade-offs regarding coverage area, signal latency, and path loss. Furthermore, we will address the technical aspects of integrating base station (BS) functionality directly onto these orbiting platforms. This includes a comparison between transparent “bent-pipe” payloads and more advanced regenerative architectures that allow onboard signal processing. Finally, this paper discusses the environmental challenges of space, such as atmospheric attenuation and Doppler shifts, which must be mitigated to ensure reliable NB-IoT performance.

### 2.1. Satellite Types

Over the past three decades, various organizations and authors have proposed classification schemes for satellites based primarily on mass, as mass directly influences the launch cost, mission complexity, and orbital deployment. These schemes have evolved alongside advances in miniaturization, leading to the proliferation of small satellite categories, such as micro, nano, pico, and femto. More recently, unified taxonomies have been suggested to harmonize disparate definitions and extend the classification to both extremely large and extremely small spacecraft. Table 1 summarizes the key classification schemes, their year of proposal, and the defining mass ranges introduced or refined.

Because NB-IoT systems primarily align with ITU recommendations, we specifically adopt the classification presented in [22], which outlines the distinctive characteristics, spectrum requirements, and appropriate radiocommunication services for nanosatellites and picosatellites. Its primary purpose was to provide a regulatory and technical framework for these emerging classes of small satellites, which were developed and launched at an unprecedented pace. The report defines nanosatellites, picosatellites, and femtosatellites based on mass, but it also highlights that from a frequency management perspective, factors such as operational lifetime, orbital uncertainty, and rapid development timelines are more critical than physical size. It compares the technical and operational characteristics of these small satellites with those of traditional larger satellites, covering topics such as launch arrangements, lack of maneuvering capability, low transmission power, and the use of commercial off-the-shelf (COTS) components. Importantly, the report identifies the radiocommunication services under which these satellites typically operate, such as the amateur satellite service, space research service, and Earth exploration satellite service, and discusses the regulatory implications, including the need for advance publication, coordination, and notification under the ITU Radio Regulations.

#### 2.1.1. Minisatellites

Minisatellites are the largest category within the “small satellite” classification, typically ranging in mass from 100 to 500 kg. They represent a bridge between traditional large satellites and smaller and more agile platforms. With a maximum bus power of approximately 1000 W and development timelines of 3–10 years, they share many operational characteristics with larger systems. Minisatellites can operate in various orbits, including GEO, MEO, LEO, and Highly Elliptical Orbits (HEOs). Their mission durations are substantial, often lasting between 5 and 10 years, and they are employed for a wide range of applications, from communications to Earth observation, with project costs typically ranging from 30 to 200 million USD.

#### 2.1.2. Microsatellites

Microsatellites occupy a mass range of 10–100 kg. They are characterized by a maximum bus power of approximately 150 W and development timelines of 2–5 years. Similar to minisatellites, they can operate in a variety of orbits (GEO, MEO, LEO, and HEO) with mission durations of 2 to 6 years. While these larger small satellites share similar programmatic and technical considerations, the primary focus of this document—and the classes that exhibit the most distinctive characteristics—are the smaller nanosatellites and picosatellites, which differ significantly in terms of development speed, launch arrangements, and operational constraints.

#### 2.1.3. Nanosatellites

Nanosatellites are defined as satellites with a mass between 1 kg and 10 kg. They represent a significant shift in satellite development, which is characterized by rapid timelines and low costs. Nanosatellites are often built using the CubeSat specification, a modular design based on 10 cm × 10 cm × 10 cm units (1U), with masses of up to 1.33 kg per unit. However, not all nanosatellites conform to this standard, and some use custom buses. Their development time is typically 1 to 3 years, with project costs ranging from 100,000 to 10 million USD. They are most frequently launched as secondary payloads on an opportunistic basis, often resulting in a lack of precise knowledge of the final orbital parameters until shortly before launch. Consequently, they primarily operate in the LEO. Nanosatellites generally lack onboard propulsion for station keeping or maneuvering. Their operational lifetime is typically 1–3 years, often limited by battery life and the use of commercial off-the-shelf (COTS) components. Transmitters are usually low-power (approximately 1 Watt), and they often use omnidirectional antennas because of the absence of complex attitude control systems. Nanosatellite missions are often divided into three main types: educational and amateur radio (often operating in amateur satellite service bands), experimental and research (for technology demonstration and proof-of-concept), and commercial (for services such as Earth observation).

#### 2.1.4. Picosatellites

Picosatellites are defined as satellites with a mass between 0.1 and 1 kg. They are even more resource-constrained than nanosatellites, with a typical bus power of only 5 W and maximum dimensions of 0.05–0.1 m. Their development is extremely rapid, and their operational lifetimes are generally less than one year. The technologies and programmatic approaches for picosatellites are similar to those for nanosatellites, with a strong emphasis on COTS components and rapid integration. An example mission timeline in the report shows a picosatellite going from a preliminary design review to launch in approximately 206 days, with a total mission lifetime (including de-orbit) of just over a year. Similar to nanosatellites, they are typically deployed as secondary payloads in LEO.

#### 2.1.5. Femtosatellites

Femtosatellites are an emerging class of satellites weighing less than 0.1 kg (100 g). They represent the extreme miniaturization of satellite technology, with bus power under 1 W, dimensions between 0.01 and 0.1 m, and development costs below 50,000 USD. Their operational lifetime is expected to be less than one year, aligning with the trend of rapid, low-cost missions for technology demonstration and educational purposes. While CubeSat specifications have been a major driver for nanosatellites, femtosatellites are at the forefront of further miniaturization and were, at the time of writing, the subject of ongoing research and development.

### 2.2. Orbits

Orbits are characterized by specific parameters, including altitude, eccentricity (how circular or elliptical the path is), and inclination (the angle relative to the equator), which together define the satellite’s coverage area, visibility from the ground, and time taken to complete one full revolution around the planet. The orbit characteristics are summarized in Table 2.

#### 2.2.1. Geostationary Earth Orbit

The GEO is a prograde, equatorial circular orbit at 35,786 km altitude, where the orbital period matches Earth’s sidereal day ($T=2\pi \sqrt{a^{3}/\mu }\approx$ 86,164 s). This yields a fixed ground position, enabling continuous coverage of approximately one-third of the globe. The fixed line-of-sight simplifies ground antennas (no tracking) and supports uninterrupted 24/7 services.

Modern GEO satellites employ High-Throughput Satellite (HTS) architectures with multiple spot beams and frequency reuse, multiplying capacity by orders of magnitude. Digital processors (transparent or regenerative) allow the dynamic in-orbit reallocation of power and bandwidth. Key applications include Fixed Satellite Services (FSS), Direct-to-Home (DTH) broadcasting, and maritime/aeronautical mobility.

Future evolution centers on software-defined payloads with digital beamforming, integration into multi-orbit networks (interlinked via optical inter-satellite links), and all-electric propulsion to increase payload mass and lifetime. Thus, GEO remains a high-capacity, persistent backbone in the evolving space infrastructure.

#### 2.2.2. Medium Earth Orbit

MEO spans altitudes of 2000–35,786 km, with orbital periods from 2 to 24 h. It offers a compromise: a larger footprint than LEO but lower latency and path loss than GEO. This makes it ideal for Global Navigation Satellite Systems (GNSSs). Major systems include GPS (≈20,200 km), Galileo (≈23,222 km), GLONASS (≈19,100 km), and BeiDou (≈21,528 km), all delivering Positioning, Navigation, and Timing (PNT) services.

MEO also supports communication constellations (e.g., O3b at ≈8062 km), providing ≈125 ms round-trip latency—better than GEO for interactive services—with fewer handovers than LEO.

Challenges include radiation exposure (Van Allen belts), requiring hardening, and orbital perturbations (Sun/Moon, Earth’s oblateness) that demand station-keeping. Deployment of a global constellation involves significant cost and logistics.

Technological advances—electric propulsion, digital transparent processors, and inter-satellite links—enhance capacity, flexibility, and resilience. In a multi-layer architecture, MEO acts as a reliable backbone for PNT and wide-area communications, complementing LEO’s low-latency access and GEO’s broadcast capabilities.

#### 2.2.3. Low Earth Orbit

LEO (160–2000 km) is the most accessible orbital zone, offering low latency (20–40 ms), global coverage via large constellations, high throughput, and resilience. Optimization of constellation geometry—plane count, satellites per plane, and offset—can reduce propagation delays by up to 35.9% [27].

Key systems exemplify this: Blue Origin’s TeraWave (5408 satellites, Q/V-band, 144 Gbps symmetrical) targets niche enterprise users [28]; Open Cosmos builds sovereign “gateway-less” mesh networks with optical ISL [29]. Integration with mobile devices (Non-Terrestrial Networks) requires emulation of rapid satellite motion and frequent handovers [30].

Space safety is critical: Starlink lowered over 4400 satellites from 550 to 480 km to reduce deorbit time and boost throughput [31]. The constellation performed 145,000 collision avoidance maneuvers in early 2025 alone [32]. The global LEO market is projected to reach US$24.1 billion by 2032 [33], with FCC authorizing 19,000 Starlink satellites and China filing for nearly 200,000 satellites [34,35].

LEO advantages include low latency, global reach, high bandwidth, cost-effective last-mile connectivity, network resiliency, and rapid innovation cycles. Applications span consumer broadband, in-flight/maritime connectivity, IoT/M2M, government/defense, first responders, and Earth observation. LEO is transforming global connectivity.

**Table 2:** Communication satellite types by orbit.

Orbit Type	Altitude	Characteristics	Typical Uses and Examples
GEO	35,786 km	Appears fixed in the sky, continuous coverage over a large area, higher latency (∼240 ms).	Television broadcasting (e.g., DirecTV), weather monitoring, and VSAT networks [35].
MEO	2000–35,786 km	Wider coverage than LEO, fewer satellites required for global coverage, balance of latency.	Global navigation systems (GPS, GLONASS, Galileo), mobile satellite systems [36].
LEO	160–2000 km	Very low latency, lower signal loss, and requires large constellations for continuous coverage.	Satellite internet (Starlink, OneWeb), Earth observation, direct-to-cell services [37].

### 2.3. Constellations

A satellite constellation is a group of artificial satellites that work together as a system to provide global or near-global coverage and continuous service. Unlike a single satellite that can only cover a limited portion of the Earth at any given time, a constellation distributes multiple satellites across different orbital planes to ensure that at least one satellite is always visible from any point on the Earth’s surface. Satellites in a constellation are carefully arranged in specific orbital patterns, often following standardized designs, such as the Walker Delta pattern, which optimizes coverage with a minimum number of satellites. A constellation is defined by parameters such as the number of satellites, number of orbital planes, inclination angle, and altitude.

The most well-known constellations include the following:

- Starlink (SpaceX) is the largest, with a multi-shell architecture: ≈1600 at 550 km (53°), ≈2800 at 540–1325 km (70–81°), and plans for 7500 at 340 km. Each satellite uses the Ku/Ka/V-band and laser ISL for a space-based mesh, delivering low-latency broadband globally [31].
- Blue Origin’s TeraWave opts for a hybrid LEO/MEO network of 5408 satellites with Q/V-band links, delivering up to 144 Gbps symmetrical—targeting only 100,000 premium enterprise users [28].
- IRIS^2^ (EU) is a sovereign multi-orbit constellation (LEO/MEO) led by SpaceRISE, providing secure connectivity for governments and critical infrastructure, reducing reliance on non-European providers [38].
- China pursues state-backed constellations: Guowang (13,000 satellites by 2035), Spacesail’s Qianfan (>15,000 by 2030), Honghu-3 (10,000), and filings for CTC-1/CTC-2 (200,000 total) to secure spectrum and orbital slots [35].

These diverse initiatives reflect a global race for LEO/MEO dominance, driven by commercial, strategic and security imperatives.

### 2.4. Hardware Integration

The concept of an onboard next-generation Node B (gNB) is central to the regenerative payload architecture for Non-Terrestrial Networks (NTNs), as defined by 3GPP (see Section 4). The technical report [39] establishes the fundamental framework for integrating satellite communications into the 5G system, distinguishing between two primary payload types. A transparent payload implements only frequency conversion and RF amplification, effectively operating as an analog RF repeater, where the waveform signal remains unchanged. In contrast, a regenerative payload incorporates full signal-processing capabilities, including demodulation/decoding, switching/routing, and coding/modulation functions onboard the satellite. This distinction positions the onboard gNB as an advanced architecture in which satellite platforms host complete or partial base station functionality, thereby enabling more sophisticated network operations in space.

The standardization journey for onboard gNB has evolved across 3GPP releases. In Releases 17 and 18, the 3GPP prioritized a transparent payload architecture to minimize payload complexity and facilitate early deployment [39]. During this period, the gNB remained on the ground with satellites acting merely as signal repeaters. However, Release 19 represents a significant milestone by introducing a full gNB onboard the satellite as the baseline regenerative payload configuration. This decision followed extensive technical discussions, where the industry evaluated alternatives such as the CU-DU split (gNB-CU on ground, gNB-DU on satellite). The full gNB approach was ultimately selected because it fully leverages all 5G RAN functionalities available since Rel-15 and natively supports inter-satellite links (ISL) via the standardized Xn interface [39].

The onboard gNB architecture has substantial technical advantages. By placing a complete gNB on the satellite, the system enables native support for inter-satellite links (ISL), allowing data to be routed directly between satellites without traversing ground gateways. This capability significantly reduces the latency of inter-satellite communications and creates a foundation for true global coverage. Additionally, the onboard gNB architecture supports store-and-forward (S&F) functionality in Rel-19, where satellites can temporarily store data when feeder links are unavailable and forward it later, thereby enhancing network resilience. From a performance perspective, regenerative payloads with onboard gNB reduce the round-trip time (RTT) for critical procedures such as random access and hybrid automatic repeat request (HARQ), as the gNB-UE interaction occurs entirely over the service link without the additional latency of feeder link propagation. This architectural evolution positions onboard gNB as a foundational element for future 6G networks, in which “data centers in the sky” and three-dimensional network integration are envisioned as key capabilities.

The deployment of a full 5G base station in a satellite payload faces a unique set of physical constraints driven by the space environment and the need for low-cost access to orbits. Unlike terrestrial base stations, space-based gNBs must operate within extremely limited power budgets, often averaging only a few tens of watts for small satellites, while managing the thermal dissipation of high-performance processing and radio frequency components. Mass and volume are equally critical, with launch costs ranging from thousands to tens of thousands of dollars per kg. Furthermore, all electronics must survive radiation effects, including the total ionizing dose (TID) and single-event effects (SEE), requiring selective hardening or mitigation. Finally, to achieve the necessary link margins for direct-to-device communication, large phased-array antennas (PAAs) are essential, introducing additional power and thermal demands. Table 3 summarizes the quantified values and requirements that shape the design of onboard regenerative NTN gNBs.

A collaborative international research effort [42] has successfully demonstrated the viability of connecting a 5G base station (gNB) to a geostationary satellite, validating the 3GPP Non-Terrestrial Network (NTN) framework for extending mobile connectivity to remote regions. In a live cross-country demonstration conducted at the World Expo 2025 Singapore Pavilion in Osaka, Japan, a research consortium comprising the Singapore University of Technology and Design (SUTD), SKY Perfect JSAT (JSAT), TMY Technology, Inc. (TMYTEK), Rohde & Schwarz, and VIAVI Solutions established the first end-to-end 5G New Radio (NR) NTN transmission between Singapore and Japan. The architecture placed the 5G base station and 5G core network emulator at a ground station in Japan, whereas the user equipment (UE) was located at SUTD in Singapore. The GEO satellite operated by JSAT acted as a bent-pipe relay, forwarding the 5G signal between the two locations without onboard processing. This configuration demonstrated that an existing GEO satellite can reliably support 5G NR standards as defined by the 3GPP, achieving real-time video-call connectivity, which is a significant advancement over traditional satellite communication systems that previously supported only text-based messaging. The demonstration also integrated an electronically steered antenna (ESA) for 5G NTN GEO communications, enabling more robust connectivity for challenging use cases, such as maritime and autonomous vehicle applications. Testing and validation of the end-to-end connectivity were performed using an NTN digital twin testbed covering LEO, MEO, and GEO orbits, which was developed jointly by Rohde & Schwarz and VIAVI. This milestone establishes a technical foundation for future extensions to MEO and LEO satellites, as well as the convergence of Terrestrial and Non-Terrestrial Networks anticipated for 6G [42].

In [43], the authors presented a gNodeB architecture that achieved full 5G New Radio (NR) functionality by combining a hardware-accelerated physical layer with a software-based upper layer stack. The VERSAL FPGA accelerates critical PHY operations—including FFT, cyclic prefix insertion/removal, and DMA-based data movement at 500 MHz—thereby offloading the multi-core CPU to handle scheduling, radio resource control, and packet processing via the OpenAirInterface (OAI) stack ported to AARCH64. This heterogeneous approach is designed to meet the stringent power, mass, and radiation constraints of space deployment while delivering full 5G new radio (NR) capabilities directly from orbit. Table 4 summarizes the key hardware and software parameters of this design, including the processing split, RF front-end configuration, timing and random access settings, and networking interfaces, all of which are aligned with 3GPP NTN specifications.

## 3. Provisioned Services and Challenges

In this section, we first introduce the basics of terrestrial cellular NB-IoT and LTE-M technologies and provide an overview of their capabilities. Then, we will explore the use cases for NTN NB-IoT systems. Finally, we conclude this section by providing an overview of the challenges faced.

### 3.1. Cellular IoT

mMTC services constitute one of the three foundational requirements of 5G cellular systems. This category is specifically designed to facilitate connectivity for an immense number of low-cost, low-complexity devices deployed across extensive geographical areas. mMTC devices are inherently characterized by (i) extended battery life (often exceeding 10 years), (ii) limited data throughput requirements, and (iii) a critical need for deep and extensive coverage. Common mMTC applications include smart meters, environmental sensors, asset trackers, and various wearable technologies.

Currently, 3GPP defines two main access technologies to serve the mMTC market, namely LTE Cat-M (LTE-M) and Narrowband-IoT (NB-IoT). LTE-M supports a broader spectrum of applications by offering superior throughput and accommodating higher communication demands, including voice services. In contrast, NB-IoT is tailored to maximize extreme coverage and cater to ultra-low-end use cases, prioritizing battery longevity and signal penetration.

These two technologies play complementary roles in the IoT ecosystem. Market projections suggest substantial adoption, with approximately 1.44 billion IoT devices expected to be connected via LTE Cat-M and NB-IoT by 2027. The key operational parameters for both NB-IoT and LTE Cat-M are summarized in Table 5.

Narrowband Internet of Things (NB-IoT), standardized in 3GPP Release 13, is the core radio access technology engineered for massive Machine-Type Communication (mMTC). Its design focuses on connecting an immense volume of low-complexity, power-constrained devices that are characterized by small, infrequent data transmissions and high latency tolerance.

NB-IoT fulfills the mMTC requirements for extreme coverage, multi-decade battery life, and massive capacity through several specialized technical features. A key advantage is its link budget, which achieves a Maximum Coupling Loss (MCL) of up to 164 dB, representing a 20 dB gain over conventional LTE, which ensures robust signal penetration into deep indoor and remote areas. Furthermore, the protocol was optimized for power efficiency, enabling a battery life of over 10 years through the use of specialized power-saving modes (PSMs). Regarding its numerology and deployment, NB-IoT employs LTE standards within a dedicated 180 kHz bandwidth, equivalent to one Physical Resource Block, and supports flexible deployment in in-band, guard band, or standalone modes. These narrow channels utilize specialized spacing, specifically 3.75 kHz for the upllink and 15 kHz for both uplink and downlink, to effectively balance coverage with spectral efficiency.

3GPP Release 14 significantly enhanced the standard by introducing Cat NB2 and Power Class 6 (14 dBm). These updates improve the energy efficiency of power-limited devices and boost their performance in dense urban settings. It also increases network flexibility by enabling paging and random access procedures on nonanchor carriers.

### 3.2. Use-Cases for NTN NB-IoT Systems

NTNs are a crucial pillar of future heterogeneous communication systems and address three pivotal operational needs. They guarantee service continuity by filling terrestrial coverage gaps and providing access to remote or inaccessible areas, ensure service ubiquity by offering network resilience and maintaining connectivity during terrestrial outages or disaster scenarios, and provide service scalability through dynamic traffic offloading to manage peak usage periods.

**Figure 2:**
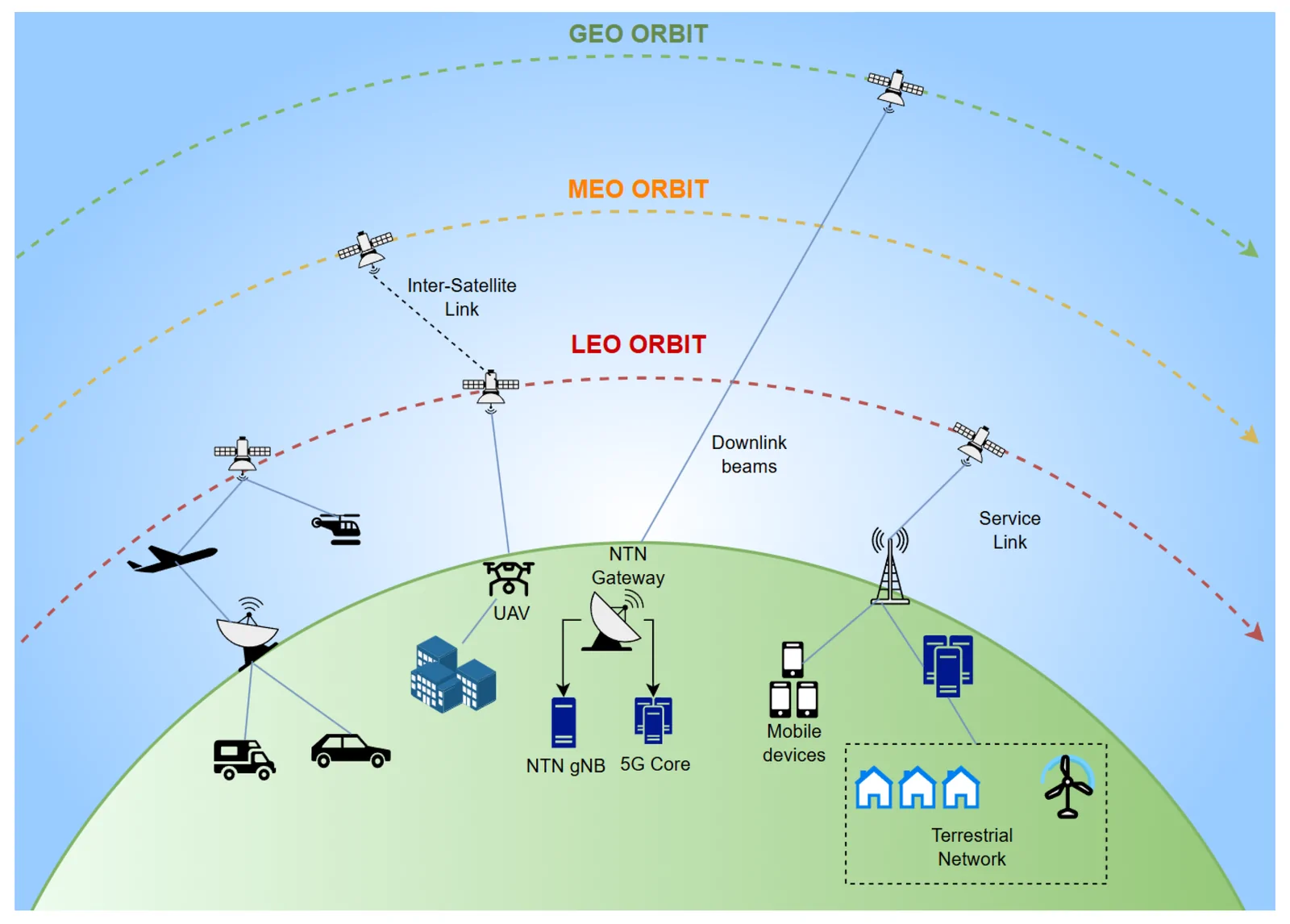
An ecosystem of NTN NB-IoT services.

The integration of NTN NB-IoT enables critical functionalities in consumer devices, such as wearables, smartphones, and vehicles, allowing for essential SOS services and two-way messaging in areas completely devoid of TN access, as shown in Figure 2. Furthermore, NTN capabilities unlock high-value applications in agriculture and farming, including precision farming and livestock tracking, particularly in remote geographical locations. This technology holds particular promise for fisheries, aquaponic farming, and diverse offshore industrial operations (e.g., offshore energy generation and mining). Consequently, the benefits offered by NTN connectivity make it a prospective candidate for monitoring remote fixed structures, such as oil rigs or isolated weather stations.

The combined utilization of both TN and NTN segments would yield substantial benefits, particularly for asset tracking and maritime applications. Assets or vehicles operating outside conventional terrestrial coverage can seamlessly rely on NTN communication. Critically, as these modules enter the vicinity of terrestrial gNBs, they can execute a seamless handover to conventional cellular connectivity. However, realizing this dynamic operational model necessitates tight integration and cooperation between TN and NTN infrastructures, which is currently an active and high-priority area of research.

Finally, NTN systems serve as an invaluable backup link for regions where infrastructure has been damaged or destroyed, ensuring communication continuity when traditional technologies are unavailable.

Random access procedure is a critical element is NB-IoT systems both TN and TNT. All mechanisms specified for TN NB-IoT systems have been replicated and enhanced by 3GPP for the NTN segment. Below, we will provide an outlook of these techniques.

#### 3.2.1. Standard 4-Step Random Access

The standard NB-IoT channel access framework, introduced in 3GPP Release 13, relies on a contention-based four-step random access procedure to transition a device from an idle state to an active connection. Once the UE establishes downlink synchronization by decoding the primary and secondary synchronization signals, it initiates access by transmitting a randomly selected preamble, known as Msg1, over the Narrowband Physical Random Access Channel (NPRACH). Upon receiving this initial signal, the base station responds with a Random Access Response, or Msg2, providing a Timing Advance command for uplink alignment, a temporary identifier, and an initial uplink resource grant. The UE then utilizes these assigned resources to transmit Msg3, which contains its unique identity and connection cause. The procedure concludes with contention resolution, or Msg4, where the base station echoes the identity of the successfully decoded UE, enabling the device to confirm its network access and commence data exchange.

Although this multistage handshake ensures reliable connectivity, its substantial signaling overhead and processing latency present significant drawbacks for battery-constrained devices sending small, infrequent data bursts.

#### 3.2.2. Early Data Transmission Regime

To streamline small-data transfers, 3GPP Release 15 introduced Early Data Transmission (EDT), allowing UEs to piggyback actual data payloads ranging from 328 to 1000 bits directly inside the Msg3 envelope. By indicating EDT intent through specialized NPRACH resources, the UE can receive a data acknowledgment and procedure termination via an RRC Early Data Complete message, eliminating the need to establish a full RRC connection.

3GPP Release 19 further expanded this concept by standardizing Contention-Based Early Data Transmission (CB-EDT) for NTN. CB-EDT removes both the preamble (Msg1) and response (Msg2) steps entirely, enabling devices to transmit data directly over the shared uplink resources. To combat severe propagation delays and potential collisions on satellite links, CB-EDT incorporates mechanisms such as Diversity Slotted ALOHA, which transmits packet replicas to guarantee high decoding success rates.

#### 3.2.3. Grant-Free 2-Step Random Access

As an alternative solution for latency-sensitive applications, 3GPP established a compressed two-step random access procedure that merges the four traditional stages into two streamlined interactions. Under this grant-free regime, the device transmits MsgA, which combines the preamble and data payload into a single transmission using Physical Uplink Shared Channel (PUSCH) resources linked directly to the chosen preamble. The base station subsequently responds with MsgB, a consolidated message performing the dual roles of resource response and contention resolution.

Bypassing the intermediate grant phase drastically reduces air-interface latency and control overhead. However, because this transmission is ungranted, it is vulnerable to co-channel interference; if multiple devices select the same preamble and identical PUSCH resources simultaneously, packet collisions occur, forcing retransmissions that can undermine the protocol’s latency advantages.

#### 3.2.4. Grant-Free Access with Fallback Option

To protect against total packet loss during high-interference scenarios, a hybrid two-to-four-step fallback mechanism was developed. In this framework, if the base station detects the preamble of MsgA but cannot decode the accompanying data payload owing to a PUSCH collision, the system dynamically shifts the transaction to the third and fourth stages of the traditional four-step process. This safety mechanism prevents complete access failure during network congestion, though the fallback transition introduces added delay and signaling overhead due to the required resource reallocation across both random access and shared data channels.

#### 3.2.5. Critical Assessment

The traditional 4-step handshake creates substantial signaling overhead and processing latency. In satellite environments characterized by long propagation delays, this multistage interaction drains the battery of constrained devices transmitting infrequent small bursts. For NTN design, the inclusion of the EDT addresses major satellite bottlenecks. By eliminating the preamble (Msg1) and response (Msg2) steps, the EDT avoids multiple round-trip delays. Furthermore, incorporating Diversity Slotted ALOHA via packet replicas may further provide a necessary countermeasure against severe propagation delays and collisions inherent to satellite links.

While 2-step grant-free access successfully minimizes air-interface latency and control overhead by combining the preamble and data payload into Msg1, its ungranted nature poses a high risk in NTN environments. The simultaneous selection of identical preambles and PUSCH resources by multiple devices triggers packet collisions. The resulting retransmissions can quickly negate the latency and efficiency gains that the scheme aims to achieve. Because ungranted 2-step transmissions are prone to failure under high interference, hybrid 2-step access with fallback to the 4-step option is an important functionality. It guarantees against total access failure when a preamble is detected, but the PUSCH payload cannot be decoded. However, designers must weigh this reliability against the penalty of added delay and signaling overhead caused by resource reallocation between the random access and shared data channels.

### 3.3. System Parameters

The 3GPP TR 36.763 on NB-IoT/eMTC support for Non-Terrestrial Networks defines a comprehensive set of satellite reference parameters for link budget analysis and system-level simulations [44]. These parameters, captured across five distinct sets (Set-1 through Set-5), span the full range of orbital regimes under consideration for IoT NTN deployment. Each parameter set reflects different satellite hardware configurations, antenna designs, and beamforming capabilities, providing a framework for evaluating NB-IoT and eMTC performance under diverse operational conditions.

Table 6 summarizes the key parameters from Sets 1–5, presenting the range of values for each parameter across all configurations. This combined representation enables a direct comparison of the trade-offs between different orbital regimes and hardware configurations, highlighting how parameter choices influence coverage, link budget, and overall system capacity.

The most significant distinction is between GEO and LEO/MEO constellations, driven by the vast difference in altitude. GEO satellites, positioned at 35,786 km, incur a maximum free-space path loss (FSPL) of approximately 191 dB, demanding substantially higher EIRP densities (53.5–59.8 dBW/MHz) and superior receiver sensitivity (G/T of 14–19 dB/K) compared to LEO systems. The narrow beamwidths of GEO (0.4–0.7°) enable highly focused spot beams but require precise pointing and limit beam count per satellite.

LEO satellites exhibit the widest parameter variation, reflecting the diversity of hardware architectures considered. Equivalent antenna aperture ranges from 0.097 m (Set-4) to 2 m (Set-1), with EIRP densities spanning 21.45–34 dBW/MHz. This variation embodies a fundamental design choice: smaller, lower-cost satellites (Set-4) produce very wide beams (1700 km diameter) providing broad coverage but at the cost of significantly reduced EIRP and G/T (−18.6 dB/K), while larger satellites (Set-1) generate narrow beams (50 km) with superior link budgets but requiring many more satellites for global coverage.

The progression from large-aperture GEO platforms to small-satellite LEO architectures (Sets 3 and 4) reflects the ongoing miniaturization trend driving LEO constellation proliferation. Set-3 (0.4 m aperture, 22.1° beamwidth) represents a balanced approach between coverage area and link budget, while Set-4 (0.097 m aperture, 104.7° beamwidth) exemplifies extreme miniaturization typical of CubeSat-based constellations. This evolution introduces new challenges in link budget closure, particularly for the uplink, where limited G/T values require careful trade-offs between coverage, capacity, and device transmit power.

### 3.4. Doppler Shift Mitigation in NTN

Doppler shift pre-compensation addresses frequency shifts that can reach several kilohertz at the 2 GHz band commonly employed for NB-IoT. The UE calculates the expected frequency shift from satellite ephemeris and its location, then offsets its uplink carrier by the inverse of this shift, ensuring the signal arrives at the satellite receiver at the nominal frequency. The 3GPP specifications delineate specific mechanisms for frequency synchronization: the UE can perform its own estimation and pre-compensation of the Doppler shift using downlink reference signals and its location, or the network can indicate the required frequency offset based on the detection of uplink signals [39]. Residual errors from spatial variation across the satellite beam are managed at the network side through reference signals and fine-tracking loops. Nevertheless, the fundamental principle remains that the UE must proactively compensate for frequency distortion prior to transmission, a requirement that presupposes accurate positioning capability.

### 3.5. Time Advance Adaptation in NTN

For timing, Time Advance (TA) is adapted for NTN operation. TR 38.821 describes two principal solutions: autonomous acquisition by the UE using its known location and satellite ephemeris, or a network-indicated approach, where a common TA component is broadcast and a UE-specific differential TA is provided by the network [39]. The network broadcasts a common delay parameter representing the minimum propagation time from the satellite to the Earth’s surface within the coverage beam. The UE calculates a differential delay based on its location within that beam and transmits earlier by the total round-trip time, ensuring the signal arrives precisely when expected. A hybrid open-loop and closed-loop approach is specified for the “RRC-Connected” state, allowing the network to refine timing via TA commands. The $K_{\mathrm{offset}}$ parameter ensures unambiguous synchronization when timing adjustments are applied, preventing temporal ambiguities in the control loop. This framework addresses the long round-trip times inherent in LEO communication, acknowledged in TR 38.811 as key design constraints affecting physical layer and protocol adaptations [45].

### 3.6. Localization and GNSS

These pre-compensation mechanisms require accurate UE positioning, establishing a fundamental coupling with GNSS. Without a location fix, the UE cannot compute the relative velocity vector required for Doppler correction or determine its distance from the satellite for differential delay calculation. The 3GPP makes GNSS capability a baseline assumption for NTN UEs, as explicitly stated in the specifications [39,44]. The standards define a “coarse location” report with approximately 2 km granularity for regulatory compliance and service management. The validity of a GNSS position fix and satellite ephemeris is managed through a network-configured timer, with radio link failure triggered upon expiration. For sporadic short transmissions, a UE may acquire a GNSS fix before accessing the network without re-acquisition during connected mode; however, longer transmissions introduce challenges such as segmented pre-compensation to handle phase discontinuity and delay drift.

GNSS reliance introduces significant implications for NB-IoT NTN devices. Integration of a GNSS receiver increases bill-of-materials cost, circuit-board footprint, and engineering effort for devices otherwise architected for low complexity. More critically, GNSS acquisition is power-intensive; for low-power IoT sensors operating on battery for years, periodic GNSS activation imposes substantial energy overhead, curtailing operational lifetime and undermining NB-IoT’s principal value proposition. Furthermore, GNSS is vulnerable in indoor environments, urban canyons, under dense foliage, or in areas with interference. The stringent timing requirements may force UEs to schedule GNSS measurement windows, suspending NB-IoT activities and introducing latency and scheduling complexity. These practical implications have been the subject of considerable discussion within 3GPP working groups, as documented in the study items [44].

## 4. Architecture and 3GPP Standardization

**Figure 3:**
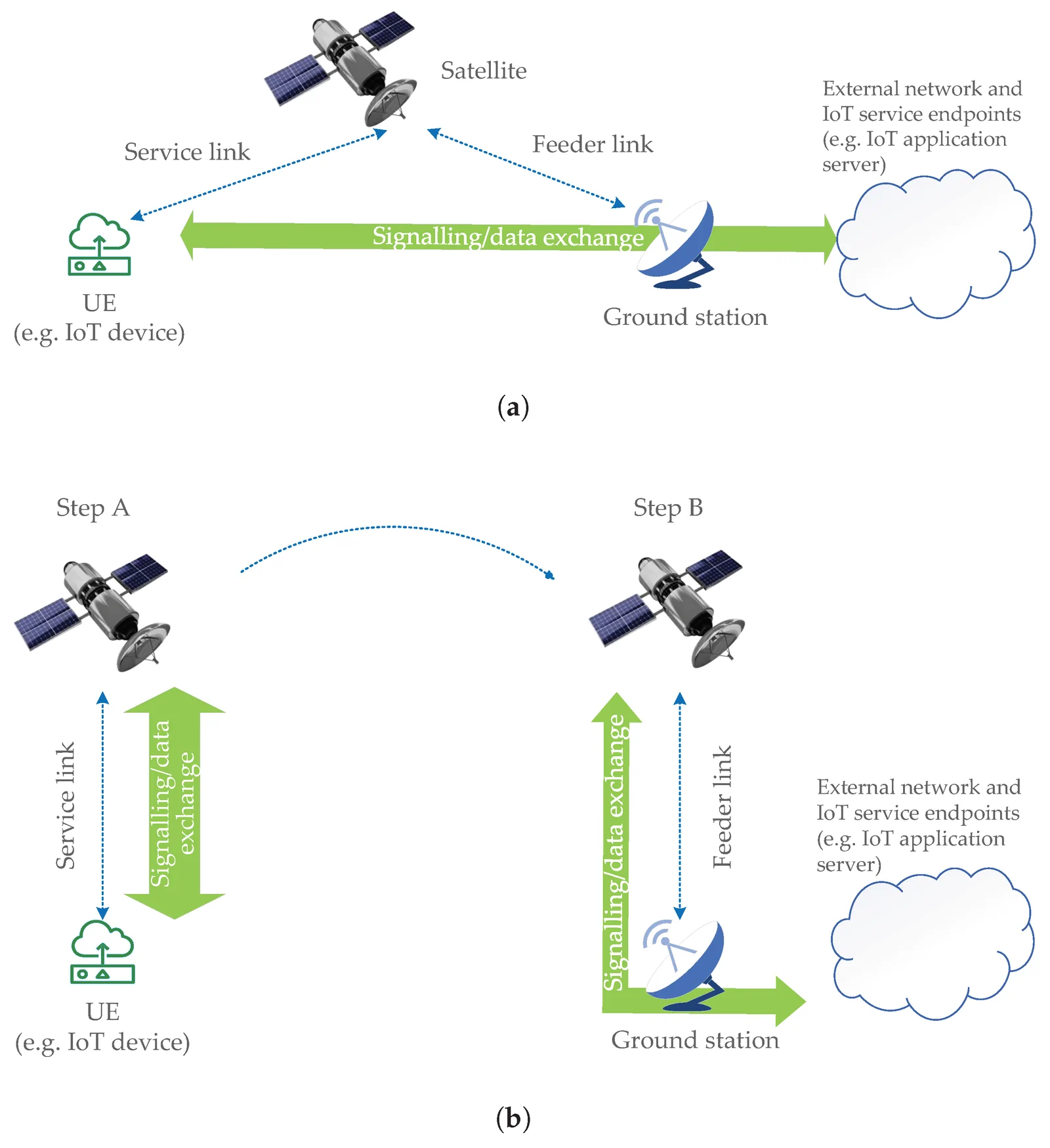
Illustration of “normal/default operation” (**a**) and “S&F operation” (**b**) modes in a 5G system with satellite access [46].

This section begins by defining the key network architectures, distinguishing between transparent “bent-pipe” payloads and advanced regenerative payloads, where the BS functionality is located directly on the satellite. It specifically highlights the evolution from the early 3GPP releases, which prioritized simpler transparent systems, to Release 19, which introduced full onboard gNB capabilities to support inter-satellite links and store-and-forward operations, as illustrated in Figure 3. Next, we cover various functional split options, such as Split Option 2, and their impact on system complexity and feeder link bandwidth requirements. This section also categorizes beam behaviors into Earth-fixed, Earth-moving, and quasi-Earth-fixed beams, and explains how these choices influence mobility management and handover frequency. A detailed roadmap of 3GPP Releases 17–19 is provided, outlining the normative specifications for timing synchronization, HARQ enhancements, and expansion into new frequency bands, such as the Ku-band. Ultimately, this section underscores the transition toward a more integrated 3D network architecture that is essential for global IoT connectivity.

### 4.1. NTN Architecture

The 3GPP framework for Non-Terrestrial Networks (NTNs) defines three principal architectures for integrating spaceborne or airborne platforms into the 5G system: transparent (bent-pipe), regenerative (with full or split gNB), and relay-like. Each architecture represents a different trade-off between onboard processing complexity, latency, and ground infrastructure’s reliance.

**Figure 4:**
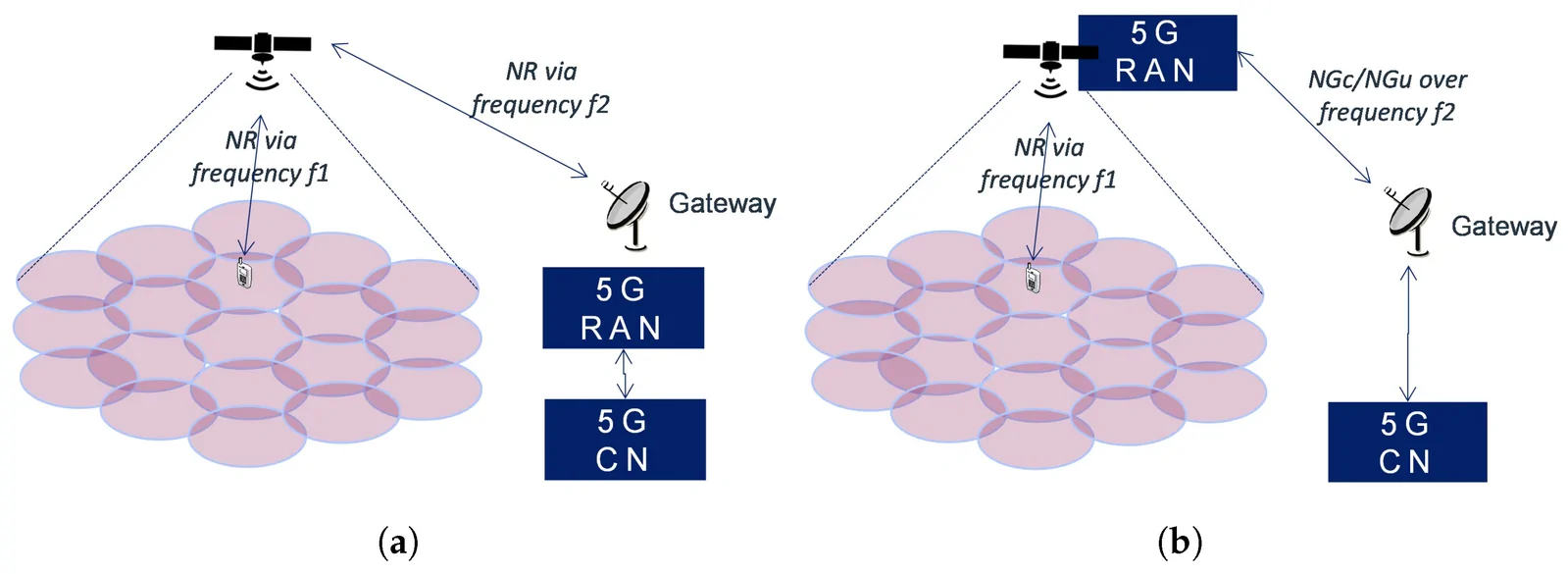
Transparent vs. regenerative payload and elliptic beam patterns [45]. (**a**) Transparent (bent-pipe) satellite/HAPS. (**b**) Non-transparent (onboard processor) satellite/HAPS.

In the transparent payload architecture, the satellite or High-Altitude Platform Station (HAPS) acts solely as an analog radio frequency (RF) repeater, as shown in Figure 4a. It performs frequency conversion, filtering, and amplification without demodulating or decoding signals. The gNB resides entirely on the ground, and the satellite merely forwards the NR-Uu waveform between the user equipment (UE) and ground gateway. As documented in 3GPP TR 38.821, the feeder link uses the same NR-Uu interface as the service link, simplifying the space segment but imposing continuous feeder link availability and adding the round-trip propagation delay of both the service and feeder links [39]. This architecture was the primary focus of the first normative NTN work in Release 17 because it minimizes payload complexity and leverages existing terrestrial gNB hardware [47]. However, transparent payloads are limited to providing services only in areas where ground stations can be positioned for concurrent feeder link and service link availability, making them less suitable for truly global coverage scenarios [48].

The regenerative payload architecture moves part or all of the gNB functionality to the platform (see Figure 4). Two variants were defined. In the full gNB onboard configuration, the entire gNB (both the Central and Distributed Units) is hosted on the satellite. The satellite then communicates directly with the 5G core (via the NG interface) and with other satellites using the Xn interface over inter-satellite links (ISLs). This variant, selected as the baseline for Release 19 after extensive industry discussions, supports store-and-forward (S&F) operations for delay-tolerant services and reduces the dependence on ground gateways [48]. According to Ericsson’s technical analysis, the full gNB onboard architecture fully leverages all available 5G RAN functionalities specified in Release 15, enabling native support for inter-satellite mobility, User Plane routing, and store-and-forward operations. In contrast, the alternative CU-DU split architecture would require proprietary inter-satellite interfaces and increase the signalling overhead due to frequent CU-DU interactions over the feeder link [49].

The second regenerative variant, the split CU-DU architecture, places the gNB-Distributed Unit (DU) on the satellite, whereas the gNB-Central Unit (CU) remains on the ground, as illustrated in Figure 4b. The two communicate over the F1 interface, which is transported over the feeder link using a satellite radio interface (SRI). The 3GPP has defined multiple functional split options, each offering different trade-offs between onboard complexity and feeder link bandwidth requirements [50]. Split Option 2 (between RLC and PDCP), which corresponds to the F1 interface, is the only split fully standardized by the 3GPP and is a strong candidate for NTN deployment. Lower splits, such as Option 6 (between MAC and PHY) or Option 7.2 (involving beamforming and resource element mapping), would place greater processing on the satellite, potentially reducing feeder link bandwidth but introducing stricter timing synchronization requirements that are challenging over satellite links with long propagation delays [39]. The choice of split option directly affects the interface bit rate requirements, maximum number of supported DUs, multi-connectivity support, and overall system complexity, with higher splits generally offering better multiplexing gain at the cost of increased onboard processing [51].

In the third configuration, the relay-like architecture, a dedicated relay node, typically a Very Small Aperture Terminal (VSAT), is introduced between the UE and satellite. The VSAT, which has larger antennas and higher transmission power than handheld or IoT devices, forwards the NR signals over the service link to the satellite. In this case, the satellite sees the VSAT as an NTN terminal rather than individual UEs. This setup is especially useful for maritime or rural applications, where a single VSAT can aggregate traffic from multiple local devices, thereby overcoming the link-budget limitations of direct satellite-to-handset communication [17].

An important architectural distinction beyond the payload type is the beam behavior. The 3GPP defines three beam categories for NTN systems: earth-fixed beams, where the coverage area remains fixed relative to the Earth’s surface (typically achieved with GEO satellites or highly controlled steerable beams); earth-moving beams, where the coverage area moves with the satellite as it traverses its orbit (common in LEO constellations without beam steering); and quasi-earth-fixed beams, which combine satellite movement with beam steering to provide intermittent coverage to fixed geographic areas [49]. The choice of beam type influences the handover frequency, cell reselection procedures, and complexity of mobility management algorithms.

Across all architectures, the service link denotes the radio interface between the UE (or the VSAT in relay-like deployments) and the NTN platform, whereas the feeder link connects the platform to the ground gateway. The choice of architecture directly affects key performance indicators such as latency, spectrum efficiency, and the ability to implement inter-satellite links. While transparent architectures have been the focus of early 3GPP Releases 17 and 18 due to their lower complexity, the standardization of onboard gNB functionality in Release 19 signals a strategic shift toward regenerative solutions. In particular, regenerative payloads with full gNB onboard enable packet switching directly in the payload using inter-satellite links without traversing the ground segment. This not only reduces latency but also enhances network resilience by allowing traffic to be rerouted in the event of a feeder link loss [48]. However, this transition remains largely prospective, with regenerative NTN NB-IoT systems yet to be commercially deployed, and the pace of adoption will depend on continued advancements in onboard processing, power efficiency, and space-qualified hardware.

From a physical layer perspective, the architecture also influences the handling of the Doppler shift, Timing Advance, and HARQ processes. In transparent payloads, the gNB on the ground must compensate for the combined Doppler of both the service and feeder links, whereas in regenerative payloads, the satellite can pre-compensate the service link, thereby reducing the complexity of the UE. These considerations are detailed in 3GPP TR 38.811 and further refined in the Release 17 specifications for NTN [45]. Additionally, regenerative payloads offer significant reductions in round-trip time (RTT) for all procedures between the gNB and UE, including random access and hybrid automatic repeat requests (HARQ), which are critical for supporting delay-sensitive applications [48].

**Figure 5:**
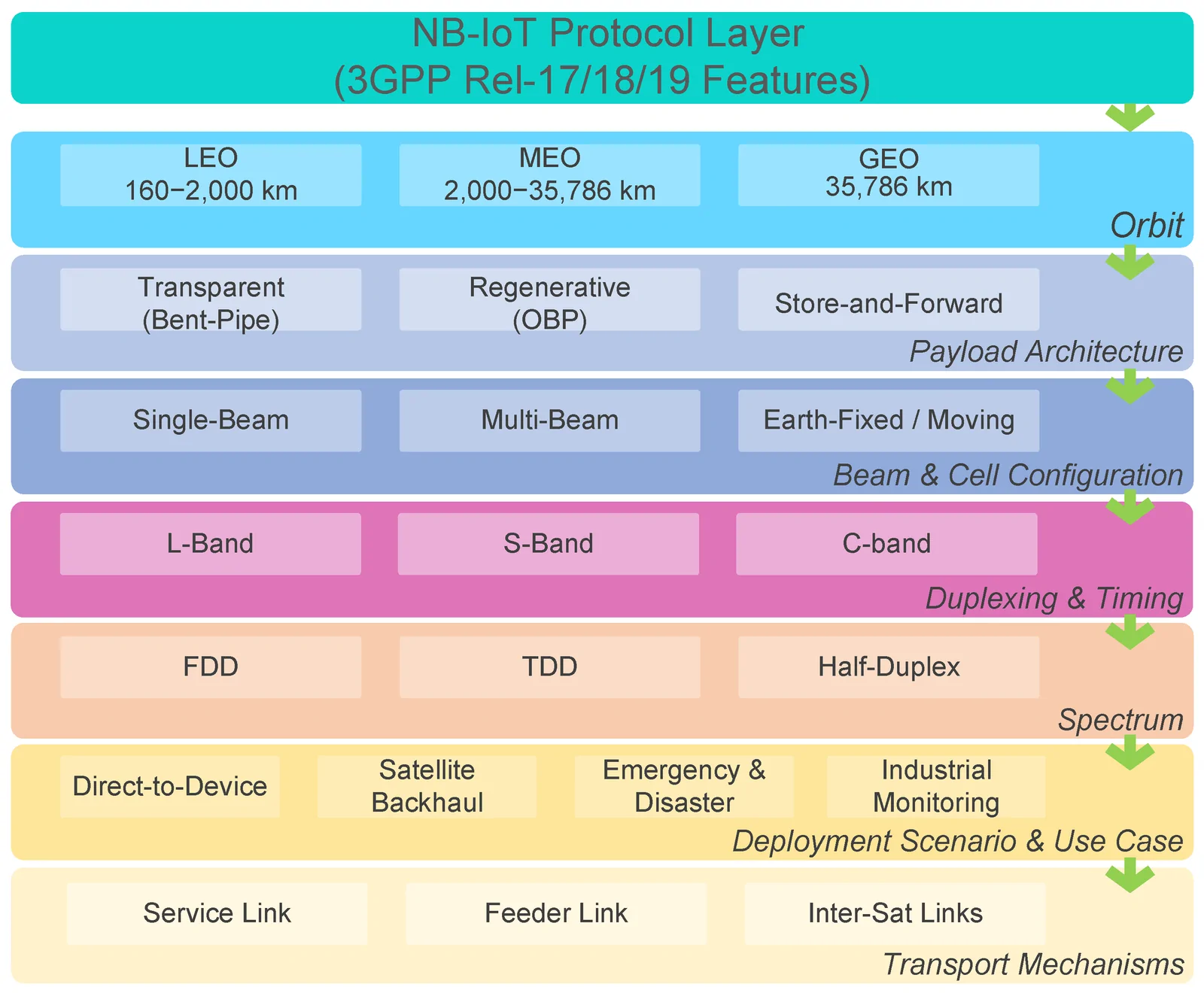
Design options for NTN NB-IoT systems.

The store-and-forward (S&F) operation represents a particularly important extension of regenerative architecture (see Figure 3b) and is expected to be fully specified in Release 19 and beyond. In the S&F mode, the satellite buffers data when no feeder link is available and forwards them when the connectivity is restored. This capability is essential for providing services in areas where ground stations are absent or during feeder link outages, and is especially valuable for IoT use cases where delay tolerance is acceptable [44]. S&F operations require enhancements to the UE paging procedure and the ability to trigger data forwarding based on contact plans that account for satellite orbits and approximate UE locations. As demonstrated in recent studies, choosing forwarding strategies based on predicted contact periods offers significant advantages over simpler strategies, although it requires a computational overhead to process orbital predictions [52]. The principle design options are illustrated in Figure 5.

Looking toward 6G, NTN architectures are expected to evolve toward fully integrated terrestrial–non-terrestrial systems, where the distinction between the two becomes transparent to the user. Multi-connectivity across different orbital regimes (LEO, MEO, GEO) and between satellite and Terrestrial Networks will enable seamless handovers and improved reliability through spatial macro-diversity [49]. The integration of regenerative payloads with edge computing capabilities, sometimes referred to as “data centers in the sky,” will further reduce latency for latency-sensitive applications and support local data processing without relying on distant ground gateways [50]. These architectural advancements are being actively studied in the context of 6G, where native NTN integration is expected from the outset, rather than being treated as an add-on to terrestrial systems.

### 4.2. NTN Standardization in 3GPP

3GPP’s work on NTN has progressed through a series of releases, each building on the findings of the previous one. The initial discussions in Release 14 (2016) were motivated by the need to extend coverage to areas lacking terrestrial cellular infrastructure, support multicast/broadcast services more efficiently, and improve network resilience during disasters. A feasibility study (TR 22.862) identified key requirements, such as service continuity between terrestrial and satellite networks and the ability to use a unified radio interface for both domains [53].

Release 15 (2018) marked the first comprehensive study item (SI) on NR to support the NTN, documented in TR 38.811. This SI examined use cases for enhanced Mobile Broadband (eMBB) and massive Machine-Type Communication (mMTC) over satellite links, adapted the 3GPP channel model to account for large propagation delays and Doppler shifts, and provided a detailed overview of deployment scenarios. It also introduced the terminology of transparent and regenerative payloads, laying the foundation for normative work [45].

The normative phase began with Release 16, which initiated two new SIs. RAN3’s SI (TR 38.821) investigates protocol enhancements for NTN, including service continuity between NTN and Terrestrial Networks (TNs), multi-connectivity, and the evaluation of transparent GEO and LEO satellite systems. SA1’s SI (TR 22.822) studied 12 specific use cases, ranging from IoT connectivity via satellite to backhaul for moving platforms, and translated them into stage 1 requirements. The findings from these SIs provided concrete proposals that guided the normative work in the subsequent release [51].

Release 17 (2022) was the first to include the normative specifications for NTN. The Work Item “Solutions for NR to Support NTN” introduced two FDD bands for satellite communication in FR1: n255 (L-band) and n256 (S-band). It also specifies enhancements for timing, synchronization, and HARQ to accommodate the long round-trip delays of satellite links. For the first time, NB-IoT and LTE Cat-M were extended to NTN, with TR 36.763 defining the necessary adaptations. This study assumed a transparent payload architecture and UEs equipped with Global Navigation Satellite System (GNSS) receivers for location acquisition [44,47].

Release 18 (2023–2024) expanded the frequency range with the introduction of band n254 (L/S-band) and increased the maximum channel bandwidth for NR NTN in FR1 to 30 MHz. A new SI on network-verified UE location mandated an accuracy of 5–10 km to satisfy regulatory requirements for emergency calls and lawful interception. The Work Item “NR NTN enhancements” improved uplink coverage through control-channel repetitions and reference-signal bundling and introduced mobility enhancements for NTN-TN and NTN-NTN handovers. In parallel, work on IoT NTN continued, with further optimization of NB-IoT random access procedures [18].

Release 19 (2025 and beyond) represents a significant shift toward regenerative payloads. The Work Item “NTN for IoT phase 3” introduces onboard gNB functionality, enabling inter-satellite links (ISLs) and store-and-forward (S&F) operations for delay-tolerant services. The new SI in SA1 explores GNSS-independent UE operation, direct UE-to-UE communication via satellite, and support for reduced-capability (RedCap) devices. Additionally, the frequency portfolio is extended to include Ku-band (10.7–14.5 GHz) operations, and the management aspects of NTN are studied to accommodate large constellations with regenerative payloads. These advancements are documented in TS 22.261 and the corresponding normative specifications [48,54].

In summary, 3GPP’s roadmap for NTN has evolved from feasibility studies in Release 14 to a full set of normative features in Releases 17–19, with a clear trajectory from transparent to regenerative architecture. The integration of NB-IoT and LTE Cat-M into NTN, together with the ongoing work on store-and-forward and inter-satellite links, underscores the central role of NTN in the 5G-Advanced and 6G ecosystems, particularly for IoT and global connectivity services [50].

### 4.3. Critical Assessment

The detailed comparison between considered architectures is shown in Table 7. The transparent or bent-pipe architecture represents the foundational approach standardized first in 3GPP Release 17, offering significant benefits such as low payload complexity and cost, minimal satellite processing, and high reliability by leveraging existing terrestrial gNB hardware. However, this architecture presents distinct drawbacks, including strict requirements for continuous feeder link availability, increased end-to-end latency, and the absence of support for ISLs. Furthermore, the ground-based gNB is forced to actively compensate for Doppler shifts affecting both the service and feeder links, and the deployment is constrained strictly to geographic regions with active ground station visibility. In contrast, the regenerative architecture featuring a full gNB onboard shifts substantial capabilities directly into space, supports inter-satellite links via the Xn interface, enables store-and-forward operations for delay-tolerant services, and offers lower service-link-only latency. This model successfully reduces the dependence on ground gateways, introduces native support for 5G RAN features, and improves the overall network resilience through in-space packet routing. Despite these operational advantages, it incurs severe disadvantages, including high payload complexity and cost, intense power consumption and thermal dissipation challenges, the necessity for radiation-hardened processing components, complex space qualification requirements, and ongoing standardization efforts.

As a middle ground, the regenerative split CU-DU architecture reduces onboard processing complexity compared to a fully onboard gNB by centralizing the Central Unit on the ground for easier management, lowering feeder link bandwidth demands, and supporting multiple satellites per a single CU. Nevertheless, this split model introduces structural trade-offs, requiring a constant F1 link between the CU and the Distributed Unit across the feeder link, generating additional latency compared to the fully onboard approach, and maintaining dependency on ground stations for CU connectivity. Additionally, selecting the appropriate split involves complex architectural trade-offs and significantly complicates CU-DU handovers.

Finally, the relay-like, VSAT-based architecture addresses severe physical constraints by overcoming link-budget limitations for handheld devices, enabling the aggregation of multiple local devices, and effectively serving areas characterized by poor direct satellite-to-handset connectivity. On the other hand, its primary disadvantages stem from introducing an additional hop through the VSAT equipment, requiring regular maintenance of this hardware, and operating under a paradigm where the satellite views the VSAT as a single consolidated terminal rather than recognizing individual UEs.

### 4.4. Future Work in 3GPP

**Figure 6:**
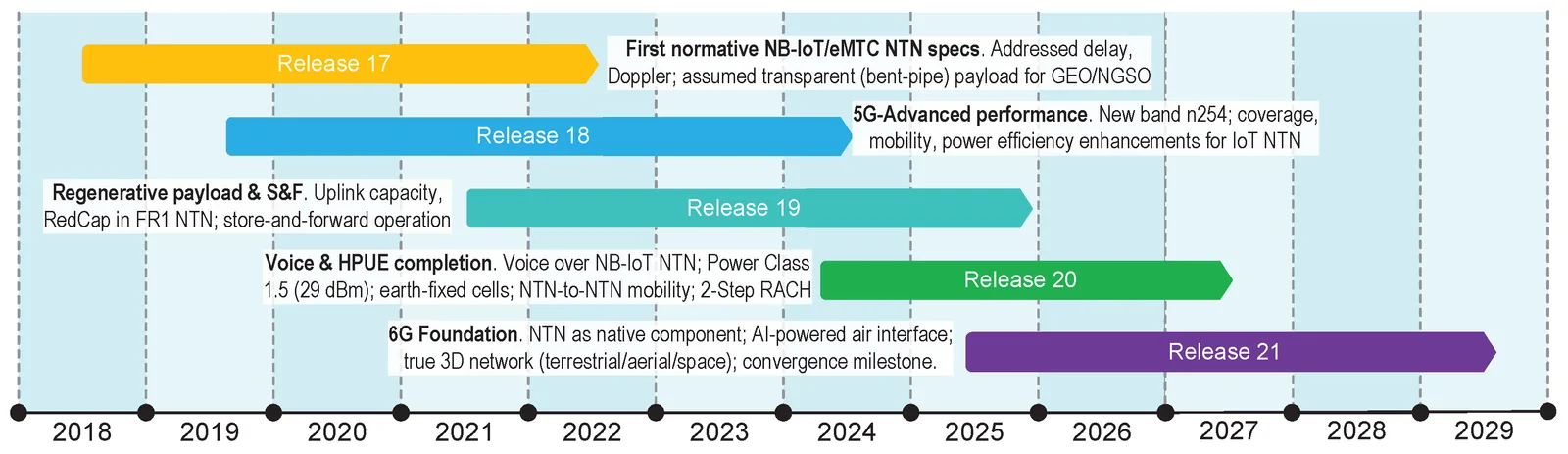
3GPP standardization progress and efforts.

A timeline for development of NTN IoT systems in 3GPP is shown in Figure 6. As 3GPP’s standardization work now moves beyond Release 19, the focus for NB-IoT NTN shifts towards completing the 5G-Advanced feature set in Release 20 and laying the foundation for a 6G-native NTN in Release 21. Release 20 (5G-Advanced Phase 3) is primarily a completion release, with the most significant new feature being the specification of IMS-based voice calls over NB-IoT via GEO satellites. A study item is actively evaluating two architectural solutions—Control Plane (CP) and User Plane (UP)—with a down-select decision expected in early 2026. Alongside voice, Release 20 will also finalize specifications for High-Power UE (HPUE) for NB-IoT NTN, specifically Power Class 1.5 (29 dBm), and is expected to incorporate other enhancements like earth-fixed cells, NTN-to-NTN mobility, and the 2-Step RACH procedure. Looking further ahead, Release 21 is set to be a pivotal “convergence milestone”, moving NTN from a 5G add-on to a native component of the 6G architecture. With a target finalization in late 2028, Release 21 will establish unified protocols for seamless interoperability between terrestrial and Non-Terrestrial Networks. It will also serve as the foundation for 6G-native NTN, incorporating AI-powered enhancements and a true 3D network architecture from the outset.

## 5. Academic Contributions

This section provides a detailed review of scholarly research focused on the design and optimization of NTN systems for the NB-IoT. It is divided into four thematic areas: architecture and design, physical layer improvements, medium access control (MAC) mechanisms, and service-oriented solutions. We highlight the transition from foundational concepts, such as transparent and regenerative payloads, to complex 6G frameworks that utilize Network Function Virtualization (NFV) and Software-Defined Networking (SDN) for flexible payload management. Research into physical layer improvements is discussed with a focus on waveform adaptation, such as the potential of Orthogonal Time Frequency Space (OTFS) modulation to handle the extreme Doppler shifts and mobility of LEO satellites. Additionally, we outline the use of artificial intelligence and federated learning to manage distributed intelligence across terrestrial and non-terrestrial layers of the network. This section serves as a technical bridge, demonstrating how academic proposals address the practical challenges of latency, synchronization, and energy efficiency identified in previous chapters. We also summarize the diverse contributions in the table to provide a clear comparison of various research methodologies and their findings.

### 5.1. Architecture and Design

In this subsection, we provide a detailed review of existing solutions for NTN architectures, ranging from foundational concepts to complex software-defined frameworks and experimental performance validation.

A foundational overview of the integration of satellite components into the 3GPP ecosystem is presented in [17]. The authors define NTN as networks employing spaceborne (GEO, MEO, and LEO) or airborne (UAS and HAPS) vehicles and classify architectures based on payload capabilities: transparent (bent-pipe) and regenerative (onboard processing). This study emphasizes that NTN in 5G is pivotal for service continuity, ubiquity, and scalability, supporting scenarios such as eMBB and mMTC in unserved areas. Rinaldi et al. detail architectural options, including direct satellite access and relay-like structures, and introduce the concept of multi-connectivity, where UE connects simultaneously to terrestrial and non-terrestrial nodes to enhance reliability.

Building on this foundation, He et al. [55] offered a broader systemic view by categorizing the ecosystem into satellite communication networks, HAPS, and air-to-ground networks. They emphasize the multilayered nature of these systems, where LEO satellites provide low latency for broadband communication, whereas GEO satellites offer wide-area broadcast capabilities. The authors also investigated the key technologies necessary for the functioning of these architectures, such as mission planning, energy management for aerial platforms, and mobility management for handling the high dynamics of non-geostationary satellites.

With the transition to 6G networks, the complexity of integrating heterogeneous layers requires a strict protocol definition. Rago et al. [56] proposed a multilayer approach detailing the integration of TN and NTN. They demonstrated how a regenerative drone, acting as a gNB, terminates protocols on board, reducing latency compared to transparent repeaters, where the gNB remains on the ground. They also investigated the deployment of Integrated Access and Backhaul nodes in NTN, where the satellite payload can serve as an IAB-Donor for terrestrial child nodes.

Tomaszewski et al. [57] propose ETHER—a unified framework for managing the complexity of the “Network of Networks” in 6G. Unlike works focused on physical connectivity, ETHER emphasizes softwarization, utilizing Network Function Virtualization (NFV) and Software-Defined Networking (SDN) to create a unified Management and Orchestration (MANO) layer. This architecture introduces a 3D infrastructure layer that combines terrestrial, aerial, and space segments coordinated by an AI-based closed-loop automation system. The authors argue for the necessity of a “Flexible Payload” concept using FPGA and container-based virtualization for the dynamic reprogramming of satellite resources.

Although theoretical architectures are well-defined, practical implementation faces significant obstacles. Baselga et al. [58] present an experimental performance analysis of transparent 5G NTN architectures, using an operational mega-constellation (Starlink) as a relay. Using OpenAirInterface (OAI), they tested three scenarios: relaying the backhaul network, midhaul network (F1 interface), and NR radio interface. Their empirical results showed that backhaul and midhaul relaying is viable with delays of approximately 50–60 ms, whereas relaying the NR signal (direct access) via a transparent payload causes critical latency issues, often exceeding one second owing to traffic overload by the RFSimulator tool. This study highlights the practical necessity of regenerative architecture for delay-sensitive procedures.

Finally, the NTN architecture is increasingly viewed as an enabler of distributed intelligence. Farajzadeh et al. [59] proposed a distributed Hierarchical Federated Learning (HFL) framework embedded within the NTN architecture. They identified the HAPS constellation as the optimal “intermediate” layer for performing distributed FL server functions owing to its balanced position between ground users and satellites. In the proposed architecture, LEO satellites act as clients alongside ground devices, and GEO/MEO satellites serve as relays for global model synchronization. This study demonstrates how the physical architecture of NTN can be leveraged to enhance privacy and computational scalability in 6G systems.

### 5.2. Physical Layer Improvements

In this subsection, we identify three main directions for physical layer improvement in Non-Terrestrial Networks: waveform adaptation, AI application, and security assurance.

Machine learning algorithms provide a powerful tool for compensating for delays and channel aging, which are critical for NTN. De Filippo et al. [60] propose using lightweight convolutional neural networks (CNNs) with an encoder–decoder architecture to predict the time–frequency channel response. The proposed method alternates between slots with pilot signals and pilot-free slots, where the channel state is predicted by the neural network. This allows for a reduction in pilot overhead and an 8% increase in effective throughput for high-order modulation schemes, although the method shows limited effectiveness for QPSK at low SNR values.

In Zhang et al. [61], deep learning methods were applied to optimize handover strategies in LEO satellite networks. The authors model the beam-switching process as a directed graph and use a CNN to extract hidden patterns from historical Reference Signal Received Power (RSRP) data. The proposed approach allows the UE to make suboptimal handover decisions by predicting the signal level of the next beam, significantly reducing the number of unnecessary switchings and the signaling load.

The unique characteristics of NTN channels (wide coverage and line-of-sight) make them vulnerable to eavesdropping. Research in this area focuses on leveraging channel properties and developing new hardware solutions for data protection. Memarian et al. [62] investigate the application of reconfigurable intelligent surfaces (RIS) installed on High-Altitude Platform Stations (HAPSs) to protect against aerial eavesdropping devices. The authors proposed an algorithm for the joint optimization of active beamforming (at the base station) and passive reflection (at the RIS) based on the fractional programming method. The results show that the RIS-HAPS system significantly increases the Secrecy Rate compared with systems without RIS or with random phases.

Review papers [63] systematize Physical Layer Security (PLS) methods in integrated satellite-terrestrial networks. The authors identified four key methods: resource allocation, beamforming, cooperative communication, and the use of physical channel characteristics (e.g., atmospheric scintillation and Doppler effect) for secret key generation.

### 5.3. Medium Access Control Mechanisms

Integrating satellite segments into the IoT ecosystem requires adapting MAC layer protocols to the conditions of extreme delays and high device density. In this subsection, we examine the proposed solutions.

Mwakwata et al. [64], in their survey, highlight that architectural changes in 3GPP releases (Release 13–16) have enabled a coverage increase of about 20 dB (Maximum Coupling Loss up to 164 dB) compared to legacy LTE technologies. These improvements are critically important for ensuring a battery life of up to 10 years; however, peak data rates remain limited to approximately 250 kb/s in the downlink and 226.7 kb/s in the uplink, which dictates strict requirements for spectral efficiency in satellite channels.

Researchers have primarily focused on the RACH procedure, where critical delays and collisions occur. Kodheli et al. [52] establish, base on a real-world testbed, that the Single User Access Time in a scenario with a GEO and transparent payload reaches 970 ms. For comparison, in Terrestrial Networks, the baseline processing delay is only approximately 12 ms. This confirms the necessity of adapting MAC layer timers, as proposed in theoretical studies such as that of [65].

To address congestion problems in LEO networks with a limited visibility window, Amatetti et al. [66] proposed the Smart Backoff algorithm. Numerical simulations showed that this method allows for an increase in the percentage of users successfully completing the access procedure by 16% compared with the standard backoff mechanism. In a scenario with a high connection density, using the standard backoff index (equal to 13) resulted in successful access for only 52% of users, whereas the proposed algorithm significantly improved this indicator.

The MT-eCD method proposed by Lee et al. [67] utilizes multi-threshold preamble detection. The results show that the algorithm maintains the PUSCH resource usage efficiency at a level above 0.92. Furthermore, the miss detection probability is less than 1%, and the false alarm probability is less than 0.1% when the SNR is above −10 dB, which complies with the 3GPP requirements.

Tuninato et al. conduct [68,69] a detailed comparison of HARQ and RLC ARQ protocols using the Land Mobile Satellite (LMS) channel model. The use of HARQ provides a gain of more than 8 dB for QPSK modulation and almost 18 dB for 256-QAM at a target BLER of $10^{-2}$. The analysis also shows that for MEO orbits, more than 32 HARQ processes are required to fill the RTT time, rendering the stop-and-wait mechanism ineffective. In LEO scenarios, the latency when using RLC ARQ can increase exponentially, reaching over 2000 ms at the maximum number of retransmissions (31 attempts), whereas HARQ maintains delays at a significantly lower level owing to a lower number of required retransmissions.

Mandawaria et al. [70] quantitatively evaluate the advantages of abandoning the resource request procedure (Grant-Free). Their dynamic resource allocation scheme demonstrated a reduction in overall latency of 15% for video traffic and 20% for IoT traffic compared with the traditional 5G NR procedure (at a target latency of 30 ms). To ensure a collision probability of no more than 10% at a packet arrival intensity of 1/s, the allocation of approximately 100 Resource Units (RU) is required.

Clazzer et al. [71] show that spectrum coexistence allows for achieving a peak throughput of 0.75 packets/slot, while rigid channel segregation limits this indicator to a level of 0.7 packets/slot. A target Packet Loss Rate (PLR) of $10^{-2}$ in the coexistence scenario is achieved at a channel load of $G_{b}=0.7$. However, the segregation scenario can maximize spectral efficiency at the cost of reducing access fairness (Jain’s index drops to 0.5).

### 5.4. Service Performance

The integration of terrestrial and Non-Terrestrial Networks supports a wide range of services, from the Internet of Things (IoT) to critical communications.

Challenges arising at the network and transport layers during the deployment of NTN into the 5G ecosystem were examined in detail by Bacco et al. [72]. This study focuses on issues related to the high latency and packet loss characteristics of satellite channels. Key topics include “Smart Gateway Diversity” to combat weather attenuation in the Q/V bands and the use of hybrid satellite-terrestrial backhaul networks.

The applied aspect of using 6G NTN for IoT was revealed by Zhou et al. [73], who proposed an architecture for ecological surveillance in remote and hard-to-reach regions. The authors identified two operating modes: satellite-assisted mode (SAM) for data collection from terrestrial sensors and satellite-dedicated mode (SDM) for direct remote sensing. A multidimensional service coverage model was developed, including three interaction modes (collection, transmission, and processing). An AI-based strategy is applied for resource management, allowing dynamic switching between local processing on the satellite and data transmission to the cloud for processing. The simulation showed an improvement in IoT service coverage of 9.99% compared to typical NTN service patterns.

While latency represents the most significant performance challenge for NTN systems compared to terrestrial deployments, throughput is also constrained by the narrowband nature of NB-IoT, which fundamentally limits peak data rates to approximately 250 kbps. Under favorable deployment conditions, the available throughput may be sufficient for many IoT applications, though practical demonstrations have reported significantly lower values (e.g., 5 kbps in the Airbus and OQ Technology trial [74]). It is also of special importance that NTN NB-IoT deployments may be suitable for state-update applications [75,76] the performance of which is conventionally measured using age-of-information (AoI) or its peak version (PAoI). The rationale is that this metric is measured at the receiver (e.g., control center located on the Earth) and is affected not only by the network latency but also by the frequency of packet generation at the source [77]. Thus, the sources can be configured such that the AoI/PAoI metrics are minimized and their values are comparable to those of terrestrial deployments.

### 5.5. AI-Driven Enhancements

Similarly to other fields, the use of ML and AI techniques also attracted attention from the academic community in context of NTN NB-IoT systems. In [78], the authors addressed the massive access congestion problem in LEO NTN IoT systems, where sporadic uplink data reports from numerous devices within a short satellite visibility window lead to high collision rates. To overcome the limitations of traditional access schemes, we propose a neural network-based algorithm designed to classify the number of colliding users and estimate their time-of-arrival (ToA) during uncoordinated random access. Evaluated under both line-of-sight (LoS) and non-line-of-sight (NLoS) conditions in suburban environments across different satellite configurations, the proposed deep learning method demonstrates significant performance benefits over conventional ToA estimation techniques, thereby enhancing access success probability for 6G NTN-IoT architectures.

A NB-IoT random access scheme based on change point detection in NTNs has been proposed by [79]. This study addresses the critical challenge of random access in NB-IoT systems operating over NTNs GNSS connectivity. The authors proposed a two-stage time of arrival (ToA) and carrier frequency offset (CFO) estimation scheme that utilizes change point detection (CPD) applied to the phase series of the received signal, backed by machine learning techniques. By preprocessing the signal and analyzing abrupt phase variations, the proposed scheme achieves high estimation accuracy under low SNR and severe Doppler shift conditions, which are typical of satellite environments. Simulation results confirm that this approach effectively performs robust random access tasks with low miss and false alarm probabilities, making it highly practical for seamless NTN-TN integration.

The work in [80] focuses on expanding wireless coverage for terrestrial fixed and mobile IoT devices in border areas and high-density urban hotspots lacking fixed infrastructure by deploying unmanned aerial vehicle (UAV)-based NTNs. To optimize the efficiency of wireless coverage and accommodate a massive number of connected IoT nodes, a deep reinforcement learning-based approach for UAV path planning and node selection was introduced. Through comprehensive simulation experiments in urban hotspot scenarios, the proposed method demonstrates its capability to deliver superior downlink rates and enhanced connectivity service performance for a greater number of IoT devices compared to traditional terrestrial NB-IoT frameworks.

Ogbodo et al. [81] propose an advanced integrated framework combining NTN (such as LEO and HAPs), UAVs, and 5G private edge networks to tackle critical rural smart agriculture challenges like limited connectivity, high deployment costs, and real-time processing demands. The architecture leverages diverse artificial intelligence paradigms, including reinforcement learning for adaptive control, federated learning for decentralized training, graph neural networks for network representation and routing, and particle swarm optimization for coordinated operations. Comparative evaluations show that this AI-driven NTN and edge framework significantly reduces energy consumption, improves latency, and lowers operational costs relative to traditional centralized systems, offering a robust solution for sustainable rural agricultural digitalization.

To conclude the review, we provide a comparative Table 8 featuring all papers.

## 6. Operational Systems and Lessons Learned

After a short introduction to the key performance indicators for mMTC services, this section covers the currently available testbeds and deployments of NTN NB-IoT systems. It then discusses the main lessons learned from these early deployments.

### 6.1. Testbeds and Deployments

The fundamental characteristics of NB-IoT distinguish it from conventional cellular technology. Operating on a licensed spectrum with a narrow bandwidth of 180 kHz, NB-IoT achieves a maximum link budget of 164 dB, providing approximately 20 dB of additional coverage compared to traditional 4G networks. This translates to the ability to penetrate three layers of concrete and reach basements, underground pipes, and other challenging environments where conventional cellular signals fail [82].

Power efficiency is perhaps the most defining characteristic of NB-IoT systems. Through power-saving mode (PSM) and extended discontinuous reception (eDRX), NB-IoT devices can achieve 5–10 years of battery life on a single AA battery, with power consumption measured in microamperes during sleep states [83]. This extreme efficiency comes with deliberate trade-offs: data rates are limited to approximately 250 kbps, latency ranges from 1 to 10 s, and mobility support is restricted to devices moving slower than 30 km/h. A single NB-IoT cell can support between 50,000 and 100,000 connected devices, enabling massive deployments in urban environments [84].

The extension of NB-IoT to satellite-based Non-Terrestrial Networks represents a significant evolution, addressing the fundamental challenge of coverage in remote and underserved areas. Multiple major developments in late 2024 and early 2026 demonstrate the accelerating commercialization of the NB-IoT NTN.

In December 2024, Mavenir and Terrestar Solutions achieved their first live NB-IoT data sessions via the Echostar T1 geostationary satellite under real-world conditions. The trials demonstrated comprehensive functionality, including network attachment, paging, ping, data sessions, and non-IP data delivery (NIDD), with continuous 24 h connectivity validation using commercial IoT modules [85]. Mavenir developed a custom Radio Access Network (RAN) interface to seamlessly integrate with TSI’s satellite ground station equipment. This milestone validated the maturity of 3GPP-standardized NB-IoT NTN technology for commercial deployment and established a dedicated testing laboratory in Montreal, equipped with Mavenir’s cloud-native RAN and Core solutions running on Amazon Web Services (AWS) Public Cloud, to accelerate partner integration.

In July 2025, Airbus and OQ Technology conducted the first flight demonstration of LEO 5G NTN connectivity to a moving drone using a licensed S band NB-IoT. The trial achieved a 5kbps throughput with 99.95% connection continuity, even during aerobatic maneuvers, including loops and spins [74]. The S-band selection proved particularly significant, offering robustness against weather conditions and forest penetration compared with the higher frequency Ku and Ka bands while maintaining compatibility with 3GPP NTN standards. According to OQ CEO Omar Qaise, licensed bands also offer advantages over unlicensed alternatives such as LoRa, in terms of reduced interference and greater protection. This demonstration marked a major step toward 5G/6G NTN support for airborne platforms in applications such as aviation, defense, and secure IoT.

In January 2026, Iridium Communications announced the successful on-air testing of Iridium NTN Direct, positioning it as the world’s first truly global, standards-based NB-IoT direct-to-device service [86]. Leveraging new 5G waveform algorithms implemented on Iridium’s software-defined LEO satellites via a simple software update, the system transmitted the first mobile-originated messages using Nordic Semiconductor’s nRF9151 module, which is a commercially available low-power cellular module. The first message sent over the service read: “To Iridium and Beyond.” Iridium plans to enter beta testing in 2026, with a commercial service launch targeted for the same year, offering 100% global coverage without requiring additional terrestrial infrastructure. Iridium NTN Direct is being developed as the world’s first truly global, standards-based NB-IoT, and direct-to-device NTN service.

Sateliot is a European satellite telecommunications company headquartered in Barcelona, founded in 2018. It has deployed the first LEO satellite constellation under the 3GPP standard (Release 17 NTN), enabling unmodified commercial NB-IoT devices to connect directly from space [87]. In May 2026, Sateliot and Turkcell successfully completed a field demonstration of 5G NTN IoT connectivity via satellite in Barcelona and Istanbul, validating standard-based 5G NB-IoT NTN connectivity over Sateliot’s LEO constellation and demonstrating its seamless integration as an extension of Turkcell’s terrestrial cellular network. The test confirmed that cellular IoT devices can seamlessly remain connected when they leave terrestrial coverage and switch between mobile and satellite networks without service interruption. Following the success of this trial, Sateliot and Turkcell plan to explore future deployments to consolidate a more connected IoT ecosystem. Sateliot also plans to launch the new generation of Tritó satellites in 2027.

Skylo runs a global Non-Terrestrial Network spanning 36 countries across 70 million square kilometers of coverage [88]. Skylo works with several GEO satellite operators, including Viasat/Inmarsat, Ligado, TerreStar, and EchoStar. In January 2026, Vodafone IoT announced a partnership with Skylo to provide NTN NB-IoT satellite connectivity services to customers. In the initial phase, the two companies will trial the technology to offer a full commercial service. Skylo and Vodafone IoT’s network cores will be integrated, enabling customers to seamlessly switch between cellular and NTN connectivity from a single Vodafone SIM. Skylo’s network orchestrates multiple satellite constellations and integrates directly into existing chipsets, modules, and operating systems. Skylo operates as a standard 3GPP NB-NTN network accessible through mobile network operator (MNO) partnerships, allowing certified devices to roam automatically between terrestrial and satellite coverage.

In February 2026, Deutsche Telekom launched the world’s first multi-orbit IoT roaming service, becoming the first network operator to offer IoT connectivity via both GEO and LEO satellites [89]. This solution ensures that IoT devices can transmit their data seamlessly and worldwide, either via terrestrial mobile networks or satellites, depending on the situation. Multi-orbit roaming was demonstrated using a commercial NB-IoT device that operates across GEO and LEO satellites and Terrestrial Networks. The solution connects Deutsche Telekom’s global IoT network (NB-IoT and LTE-M) to satellite services from several partners. Skylo provides coverage in GEO, whereas Sateliot and OQ Technology handle radio connectivity with LEO satellites. Additionally, in the second half of 2026, Iridium’s NTN Direct will be available to DT’s business customers for IoT applications. The service has been validated on Nordic Semiconductor’s nRF9151, the first 3GPP-compliant cellular IoT module to support terrestrial NB-IoT/LTE-M and NB-NTN over both GEO and LEO.

OQ Technology is a global pioneer in satellite 5G IoT and Direct-to-Device (D2D) connectivity [90]. In December 2025, the company announced a major milestone: a successful end-to-end NB-IoT connection and IoT data transmission from Nordic Semiconductor’s nRF9151 low-power cellular IoT module to its LEO satellite constellation. Unlike other NTN demonstrations that rely on third-party protocol stacks, OQ’s achievement is powered entirely by its 3GPP-compliant NTN NB-IoT RAN stack and a fully operational and integrated 5G core network. This vertical integration gives OQ control over the performance, reliability, and service optimization across the full network chain. The test verified that the nRF9151 IoT module could connect directly to OQ’s satellites without hardware modifications, demonstrating the readiness of low-power, standard-compliant NTN IoT for broad commercial deployment. In January 2026, OQ Technology partnered with Monogoto to integrate its 3GPP-compliant 5G NTN NB-IoT service into Monogoto’s platform.

Table 9 summarizes representative commercial and trial deployments of NB-IoT, ranging from large-scale terrestrial smart metering to emerging Non-Terrestrial Network (NTN) demonstrations.

### 6.2. Lessons Learned

The applicability of NB-IoT NTN spans scenarios in which terrestrial coverage is economically or geographically infeasible. For mobile network operators, NTN integration enables coverage extension without capital-intensive tower construction, offering an incremental opportunity to eliminate coverage gaps and enhance subscriber retention [48]. This technology enables single hardware platforms to support both terrestrial and satellite connectivity, thereby reducing device complexity and cost.

Several challenges have emerged in deployed NB-IoT systems. In terrestrial deployments, signal quality remains heavily dependent on environmental factors, with urban areas experiencing fluctuations owing to interference and mobility, whereas rural areas face weaker but more consistent signals [96]. The fallback to 2G and 4G networks implemented in the Airtel deployment acknowledges that NB-IoT coverage, despite its theoretical advantages, remains subject to operator network density and deployment decisions.

Synchronization impairments present the most significant technical hurdle for NTN deployments. The Doppler shift, carrier frequency offset (CFO), and symbol timing offset (STO) induced by satellite movement require sophisticated compensation algorithms. Even with GNSS-based pre-compensation, residual errors can generate inter-carrier interference and phase rotation that degrade the decoding performance, particularly for higher orbital altitudes and longer data payloads [97]. The long propagation delays inherent in GEO satellite links necessitate adaptations to the standard timing alignment and HARQ processes [98].

Spectrum licensing presents another challenge: while the licensed spectrum provides protection from interference and ensures predictable performance compared to unlicensed alternatives such as LoRa, it requires coordination with mobile network operators and adherence to regional spectrum regulations. The S-band approach used by Airbus and OQ Technology offers a pathway that aligns with the 3GPP NTN standards while providing favorable propagation characteristics [74].

Several key lessons have emerged from the deployed NB-IoT systems:

1. The importance of fallback mechanisms is evident: the Airtel deployment’s inclusion of 2G and 4G fallback recognizes that NB-IoT coverage may not be universally available and that uninterrupted service requires redundancy [99]. Second, early demonstrations have shown the feasibility of using commercial, standards-based hardware for NTN NB-IoT connectivity. Both the Mavenir/Terrestar GEO trial [85] and the Iridium NTN Direct test [86] successfully utilized commercially available modules without modification, suggesting that existing hardware may be adaptable for satellite-based IoT applications. However, further validation across a wider range of deployment scenarios and longer operational periods is needed to confirm long-term reliability and performance.
2. The flexibility of software-defined infrastructure has proven critical to NTN advancement. Iridium’s activation of NB-IoT capabilities via a software update to its existing LEO constellation demonstrates how satellite networks can evolve without hardware replacement [86]. Similarly, Mavenir’s cloud-native RAN and Core solutions running on public cloud infrastructure enabled rapid deployment and testing [85].
3. The trade-off between coverage and capacity requires careful engineering. Higher-altitude platforms provide larger coverage footprints but suffer from reduced signal strength and network capacity owing to increased path loss [100]. The GEO approach adopted by Terrestar offers stable coverage but with higher latency, whereas Iridium’s LEO constellation provides lower latency but requires more complex handover management.
4. The ecosystem approach to NB-IoT deployment—encompassing chipset makers, module manufacturers, network operators, and application developers—has proven essential to success. The collaborations between Nordic Semiconductor and Iridium and between Mavenir and Terrestar illustrate that commercialization requires alignment across the entire value chain [85,101].

The trajectory of NB-IoT deployments points toward the continued expansion of both terrestrial and non-terrestrial applications. As a foundational component of the 5G mMTC roadmap, NB-IoT will receive ongoing standardization support and integration with emerging technologies, including edge AI, post-quantum cryptography, and hybrid connectivity architectures. The technology’s inclusion in 3GPP Releases 17 and 18 ensures its evolution alongside 5G and future 6G networks [48]. With commercial NB-IoT NTN services expected to be launched in 2026 by multiple operators, the technology is positioned to deliver on its promise of truly ubiquitous, low-power connectivity for the Internet of Things.

Table 10 summarizes the key performance values reported for NB-IoT in commercial deployments, field trials, and analytical studies. The metrics cover the fundamental aspects of NB-IoT operation: reliability (packet delivery ratio), coverage extension beyond LTE-M, device capacity per cell, power consumption with mobility, latency under load, and signal quality dependence on the environment.

### 6.3. Challenges and Future Developments

#### 6.3.1. Challenges of the Current Implementations

In spite of significant step forwards made in latest 3GPP releases, there are several key challenges that remain unresolved or present ongoing design hurdles for NTN NB-IoT systems, spanning propagation constraints, random access inefficiencies, hardware limitations, and architectural bottlenecks, traffic support.

Regarding propagation and link constraints, transparent (bent-pipe) architectures force ground-based gNBs to continuously and actively compensate for significant Doppler shifts affecting both service and feeder links. Furthermore, both transparent and split CU-DU architectures remain heavily dependent on continuous feeder link availability and constant F1 connectivity between the Central Unit and Distributed Unit over the satellite link, which strictly limits deployments to geographic areas with active ground station visibility. Long orbital distances also introduce high end-to-end propagation delays, hindering real-time responsiveness and complicating latency-sensitive operations.

Random access procedures introduce persistent bottlenecks, as traditional 4-step mechanisms generate substantial signaling overhead and processing latency that severely drain the batteries of constrained NB-IoT devices. Although 2-step grant-free schemes and contention-based EDT attempt to bypass these delays, they remain highly vulnerable to co-channel interference and packet collisions when multiple devices simultaneously select identical preambles and shared PUSCH resources. While hybrid fallback mechanisms successfully prevent total access failure during such collisions, transitioning back to multi-step processes incurs secondary penalties, introducing extra delay and signaling overhead due to cross-channel resource reallocation.

Onboard hardware and power constraints continue to challenge fully regenerative architectures featuring an onboard gNB, which suffer from high payload complexity, elevated financial costs, and incomplete standardization. Operating advanced gNB processing components in space creates intense power consumption and thermal management hurdles, while the necessity for specialized, radiation-hardened processing components demands complex and expensive space qualification procedures.

Broader architectural and deployment bottlenecks persist across various models. Split CU-DU architectures significantly complicate handovers and tie system performance tightly to ground station connectivity. Similarly, relay-like (VSAT-based) configurations introduce an additional network hop and treat the VSAT system as a single unified terminal rather than recognizing individual UEs, while also imposing ongoing hardware maintenance requirements.

Finally, the topic that was totally overlooked so far is traffic support in NTN NB-IoT systems. According to ITU-R specifications [107,108], 5G NB-IoT systems must support asynchronously generated traffic, where each UE generates a 32-byte packet every two hours. In LEO-based NB-IoT systems characterized by intermittent connectivity [109] which is typical for early phase deployment, a satellite speed of approximately 7.8 km/s causes the Earth-coverage footprint to move at virtually the same rate. Consequently, even when devices operate asynchronously, entering a moving beam footprint can lead to batch traffic arrivals. Depending on the interplay between packet generation rates and uninterrupted connectivity durations, a portion of the load still arrives asynchronously, a characteristic shared by MEO systems despite their longer connectivity windows.

In contrast, GEO systems feature stationary coverage, preserving the original structure of the traffic arriving at the base station. Nevertheless, the nature of this traffic can deviate from the standard ITU-R definitions. Modern applications, such as smart electricity metering, frequently induce synchronous operations because of the use of connection-oriented protocol suites, such as DLMS/COSEM or TCP/TLS [110]. Because these applications are actively managed and regularly queried by remote application servers, air-interface traffic in GEO-based NB-IoT systems ultimately results in a mixture of asynchronous and batch arrivals. The effects have been observed in terrestrial deployments and may become even more pronounces in NTN ones [2,111].

#### 6.3.2. The Preliminary 6G Vision

Initial academic research and international standardization agendas for 6G are actively laying the structural groundwork for advanced NTN and massive IoT ecosystems. At the global regulatory level, the ITU-R established the foundational vision under Recommendation ITU-R M.2160 for the IMT-2030 framework, which prioritizes key usage scenarios, such as ubiquitous connectivity, massive communication, sustainability, and resilience. This agenda has been further shaped by ITU-R Working Party 5D through its specialized draft reports on minimum technical performance requirements for IMT-2030, offering a unified basis to evaluate 6G radio interfaces and satellite integration.

Concurrently, the 3GPP standardization roadmap has established a direct bridge from 5G Advanced to 6G. Building upon the initial satellite integrations of earlier cycles, 3GPP officially initiated Release 20 to drive exploratory technical studies on 6G, including targeted Work Items assessing NTN operation without GNSS reliance and ambient IoT integrations, while paving the way for normative specifications under Release 21, with commercial deployments envisioned around 2030.

Within these evolving standards agendas, the technical outline for NTN NB-IoT and massive connectivity centers on a unified three-dimensional architecture integrating LEO, MEO, and GEO satellite layers, with a strong architectural shift toward regenerative payloads to minimize end-to-end latency and reduce reliance on ground gateways. To overcome severe satellite propagation constraints, high Doppler shifts, and massive access congestion, academic and pre-standardization proposals have emphasized AI-enabled non-orthogonal random access, advanced ToA estimation, and robust change-point detection algorithms. Furthermore, upcoming 6G frameworks mandate GNSS-free operational capabilities, AI-native radio resource management to intelligently handle rapid beam footprint mobility and mixed batch-asynchronous traffic patterns, and strict, quantitative energy-efficiency metrics aligned with long-term global sustainability targets.

### 6.4. Economic Considerations

When comparing NTN NB-IoT to traditional terrestrial NB-IoT, several distinct economic challenges emerge, primarily revolving around connectivity pricing, hardware expenses, and infrastructure investments. Unlike Terrestrial Networks that benefit from dense, pre-existing cellular infrastructure allowing for inexpensive, high-volume data transmission, satellite-based NB-IoT suffers from a much higher cost per kilobyte owing to the immense complexity of delivering signals across orbital distances. Consequently, ongoing monthly connectivity pricing for NTN NB-IoT can be significantly higher, often multiple times more expensive per device compared to standard terrestrial options, making it economically viable only for ultra-low data volumes, such as tiny payloads sent infrequently.

Furthermore, device economics are heavily impacted by specific hardware requirements. Deploying NTN NB-IoT requires specialized Release 17-conforming modems, chipsets, and occasionally external antennas or physical modifications to overcome severe path losses and strict line-of-sight constraints, which collectively increase the overall bill of materials (BoM) and installation costs for end users. On the supply and infrastructure side, the financial barrier to entry is immense, requiring heavy capital expenditure to develop, launch, and operate satellite constellations in harsh space environments, where inaccessibility means hardware repairs or physical troubleshooting are virtually impossible. While NTN NB-IoT fills a crucial gap for remote tracking and provides cost advantages over legacy proprietary satellite solutions for tiny data packets, its financial structure cannot match the low-cost scalability of terrestrial cellular networks as soon as data usage or message frequency begins to scale up.

## 7. Conclusions

The integration of NB-IoT NTNs marks a pivotal shift toward achieving ubiquitous global connectivity. By extending 5G services to satellite and aerial platforms, the industry is addressing the 85% of the Earth’s surface that remains beyond the reach of conventional terrestrial infrastructure. This evolution is particularly critical for the provisioning of mMTC services in remote sectors such as maritime logistics and forestry, where the economic burden of ground-based stations is prohibitive.

A key technical trajectory identified in this study is the transition from transparent “bent-pipe” architectures to advanced regenerative payloads. While early deployments focused on feasibility and leveraging existing ground hardware, future 5G-Advanced and 6G systems will increasingly rely on onboard processing and inter-satellite links to reduce latency and enhance network resilience. These advancements, supported by ongoing 3GPP standardization across Releases 17–19, are essential for overcoming the unique physical layer challenges, such as extreme Doppler shifts and long propagation delays, inherent in satellite communication.

Ultimately, the successful commercialization of NTN NB-IoT depends on a coordinated ecosystem that encompasses satellite operators, chipset manufacturers, and regulatory bodies. Early field trials have validated the use of commercial, standards-based hardware and the flexibility of software-defined infrastructure to enable rapid network evolution. As global constellations such as IRIS^2^ and Guowang continue to be deployed, the lessons learned from current intermittent connectivity and high capital expenditures will guide the development of more efficient, scalable, and resilient IoT networks in the next decade. 

## Figures and Tables

**Table 1:** Satellite classification schemes and their contributions.

Scheme	Year	Specifics
Sweeting [19]	1991	Initial mass-based classification: large (>1000 kg), small (500–1000 kg), mini (100–500 kg), micro (10–100 kg), nano (<10 kg).
Konecny [20]	2004	Refined classification: added pico (0.1–1 kg) and femto (<0.1 kg) classes; retained large, medium, mini, micro, nano.
Kramer & Cracknell [21]	2008	Merged mini and medium into 100–1000 kg range; maintained micro (10–100 kg), nano (1–10 kg), pico (0.1–1 kg), and femto (0.01–0.1 kg).
ITU-R [22]	2014	Defined nanosatellites (1–10 kg), picosatellites (0.1–1 kg), femtosatellites (≤0.1 kg) with emphasis on spectrum requirements; also included mini (100–500 kg), and micro (10–100 kg) satellites.
NASA [23]	2015	SmallSat definition: satellites with mass below 180 kg; further divided into mini (100–180 kg), micro (10–100 kg), nano (1–10 kg), pico (0.01–1 kg), femto (0.001–0.01 kg).
Wekerle et al. [24]	2017	Small satellite classification: large (>500 kg), mini (101–500 kg), micro (11–100 kg), nano (1–10 kg), pico (<1 kg).
FAA [25]	2018	Payload classification for launch vehicles: extra heavy (>7100 kg), heavy (5401–7000 kg), large (4201–5400 kg), intermediate (2501–4200 kg), medium (1201–2500 kg), small (601–1200 kg), mini (201–600 kg), micro (11–200 kg), nano (1.1–10 kg), pico (0.09–1 kg), femto (0.01–0.1 kg).
Botelho & Xavier [26]	2019	Unified taxonomy with 10 classes based on powers of 10: mega, hecto, deca, protypo, mini, micro, nano, pico, femto, gram. Integrates previous schemes and adds size descriptors.

**Table 3:** Physical constraints for onboard NTN gNB.

Parameter	Requirement
Power & Thermal	
Orbital average power	as low as 38 W (microsatellite)
Peak payload dissipation	∼20 W (X-band transmitter, 26% efficiency)
Thermal control precision	<±0.15 °C (with 9.3 W avg. power)
Orbit temperature variation	4 °C to 9 °C (passively controlled small satellite)
Size, Weight, and Power	
Launch cost	$10,000–$1000 per kg (to LEO/GTO)
Payload mass (low-SWaP)	<10 kg
Payload mass (high-SWaP)	∼150 kg
Payload power (low-SWaP)	<30 W
Payload power (high-SWaP)	∼400 W
Radiation Hardening	
Total ionizing dose (TID)	100 krad (space-grade); 30–50 krad (rad-tolerant)
Single event effects (SEE)	up to 75 MeV·cm^2^/mg (rad-hard controllers)
Hardening standards	ECSS-Q-ST-60-15C, MIL-HDBK-533, etc. [40]
Phased Array Antenna	
Number of elements	∼100 (typical high-throughput system) [41]
System power consumption	<1 kW (100-element PAA) [41]
Achievable performance	SNR and beam crosstalk >20 dB [41]

**Table 4:** Onboard 5G gNB parameters [43].

Parameter	Description
Processing platform	AMD Xilinx VERSAL AI Core (space-grade) + multi-core CPU
PHY acceleration	FPGA-based FFT, coding, rate matching, DMA at 500 MHz
Higher layers	OpenAirInterface (OAI) stack ported to AARCH64
Processing split	PHY–MAC split with PCIe interconnect
Transceiver	Analog Devices AD9082 MxFE (16-bit 12 GSPS DAC, 12-bit 6 GSPS ADC)
Interface to FPGA	JESD204B/C (up to 24.75 Gbps/lane, 8 lanes each direction)
On-chip DSP	Bypassed (direct connection to converter cores)
NTN FR1 bands	3GPP Bands 254, 255, 256 (FDD)
Channel bandwidth	Up to 50 MHz (Ku-band trials); 5–10 MHz typical
cellSpecificKoffset (GEO)	478 slots
ta-Common (GEO)	≈29,319,745
K2 (LEO)	46 slots
prach_ConfigurationIndex	98
preambleReceivedTargetPower	−118 dBm
preambleTransMax	6 (64 preamble transmissions)
pMax (UE max transmit)	20 dBm
System power consumption	Under evaluation
Xn interface	Supported for inter-satellite links
NG interface	Connects to AMF on ground
Test platform	Evaluation boards (VERSAL, AD9082)
Validation	Digital twin testbed (Rohde & Schwarz/VIAVI)

**Table 5:** NB-IoT and LTE Cat-M parameters.

	Unit	Cat NB1	Cat NB2	Cat M1	Cat M2
3GPP Release	–	R13	R14	R13	R14
Licenced Spectrum	Y/N	✓	✓	✓	✓
Frequency	MHz	700–2100	700–2100	700–2600	700–2600
Bandwidth	MHz	0.2	0.2	1.4	5
Payload size	Bytes	<1600	<1600	<8188	<8188
MCL	dB	164 dB	164 dB	155.7 dB	155.7 dB
EIRP	dBm	<23	<23	<23	<23
Tx power consumption	mA	240	240	360	360
Rx power consumption	mA	46	46	70	70
PSM	µA	<3	<3	<8	<8
Battery lifetime	years	>10	>10	>10	>10
UL data rate	kb/s	0.3–62.5	0.3–159	375, 590 (HD) 1000, 3000 (FD)	2625 (HD) 4000 (FD)
DL data rate	kb/s	0.5–27.2	0.5–127	300, 800 (HD) 800, 1000 (FD)	2275 (HD) 4000 (FD)

**Table 6:** 3GPP satellite parameters for IoT NTN link budget analysis (Sets 1–5) [44].

Parameter	GEO	LEO-600	LEO-1200	MEO-10000
Satellite altitude	35,786 km	600 km	1200 km	10,000 km
Payload type	Transparent			
Frequency band	S-band (∼2 GHz)			
DL Transmissions				
Equivalent antenna aperture	12–22 m	0.097–2 m	0.4–2 m	1.5 m
Satellite EIRP density	53.5–59.8 dBW/MHz	21.45–34 dBW/MHz	28.3–40 dBW/MHz	45.4 dBW/MHz
Satellite Tx max gain	45.5–51 dBi	11–30 dBi	16.2–30 dBi	28.1 dBi
3 dB beamwidth (HPBW)	0.4011–0.7353°	4.4127–104.7°	4.4127–22.1°	6.5°
Satellite beam diameter	250–459 km	50–1700 km	90–470 km	1140 km
UL Transmissions				
Equivalent satellite aperture	12–22 m	0.097–2 m	0.4–2 m	1.5 m
G/T (Figure of Merit)	14–19 dB/K	−18.6 to 1.1 dB/K	−12.8 to 1.1 dB/K	3.8 dB/K
Satellite Rx max gain	45.5–51 dBi	11–30 dBi	16.2–30 dBi	28.1 dBi
Beam Geometry				
Central beam center elevation	12.5–20.9°	30–90°	30–46.1°	90°
Central beam edge elevation	2.3–12.5°	23.8–30°	22.2–30°	81.6°
Link Budget				
Max FSPL (central beam edge)	190.6–190.8 dB	159.1–160.4 dB	164.5–165.8 dB	178.5 dB
Max satellite-UE distance	∼40,581 km	∼1076 km	∼3131 km	∼10,042 km

**Table 7:** Comparison of NTN architectural options.

Architecture	Advantages	Disadvantages
Transparent (Bent-Pipe)	Low payload complexity and cost Leverages terrestrial gNB hardware Minimal satellite processing, high reliability Standardized first in 3GPP R17	Requires continuous feeder link availability End-to-end latency No support for ISL gNB must compensate for Doppler of service and feeder links Limited to areas with ground station visibility
Regenerative (Full gNB Onboard)	Supports ISL via Xn interface Enables S&F operation for delay-tolerant services Lower latency (service link only) Reduced dependence on ground gateways Native support for 5G RAN features Enables packet routing in space, improving resilience	Payload complexity and cost Power consumption and thermal dissipation Radiation-hardened processing components Complex space qualification Standardization ongoing
Regenerative (Split CU-DU)	Reduces onboard processing compared to full gNB Centralized CU on ground for easier management Lower feeder link bandwidth Supports multiple sats per CU	Requires constant F1 link between CU and DU over feeder link Additional latency compared to full gNB onboard Depends on ground stations for CU connectivity Split selection involves complex trade-offs CU-DU handovers more complex
Relay-Like (VSAT-based)	Overcomes link-budget limitations for handheld devices Aggregation of multiple local devices Can serve areas with poor direct satellite-to-handset connectivity	Additional hop (VSAT) Maintenance of VSAT equipment Satellite sees VSAT as terminal, not individual UEs

**Table 8:** Summary of reviewed papers.

Reference	Main Focus	Performance Study
Network Architecture		
Rinaldi et al. [17]	Defines NTN (GEO/MEO/LEO, UAS/HAPS); transparent and regenerative architectures; multi-connectivity.	Continuity and scalability for eMBB/mMTC in remote areas.
He et al. [55]	Multilayered systems, mission planning, and mobility management for NGSO satellites.	LEO for low-latency broadband; GEO for broadcasting.
Tomaszewski et al. [57]	ETHER framework: SDN/NFV for unified management; Flexible Payload (FPGA).	AI-based automation of 3D (ground/air/space) infrastructure.
Baselga et al. [58]	Experimental analysis of Starlink; justification for regenerative solutions.	Backhaul relaying (50–60 ms) vs. critical delays (>1 s) for NR.
Zhou et al. [73]	SAM/SDM modes for ecological monitoring.	9.99% improvement in IoT coverage vs. typical patterns.
MAC & Protocol Design		
Mwakwata et al. [64]	3GPP Rel. 13–16 changes; 20 dB coverage boost (MCL 164 dB).	10-year battery life for IoT with ∼250 kbps peak rates.
Kodheli et al. [52]	RACH analysis: 970 ms access time for GEO satellites.	Comparison of NTN delays vs. terrestrial (∼12 ms).
Tuninato et al. [68]	Comparison of HARQ/RLC; need for >32 HARQ processes for MEO.	8–18 dB HARQ gain; prevents exponential delay growth (2000 ms).
Zhou et al. [73]	DRL for dynamic edge (satellite) vs. cloud switching.	9.99% improvement in IoT coverage vs. typical patterns.

**Table 9:** Technical characteristics of NTN NB-IoT deployments (standard NB-IoT bandwidth of 200 KHz used by all).

Deployment	Orbit	Frequency	Terminal Type	Source
Sateliot	LEO (SSO)	S-band (n256): UL 1980–2010 MHz, DL 2170–2200 MHz	Standard 3GPP NB-IoT modules	[91]
Skylo (via Viasat/Inmarsat/EchoStar)	GEO	L-band (n255): 1525–1660.5 MHz; S-band (n256): 1980–2200 MHz; Band 23	NB-IoT/LTE-M modules (e.g., Nordic nRF9151, Sony Altair ALT1250)	[92]
OQ Technology	LEO	S-band (LTE Band 65, ∼2 GHz); 60 MHz MSS S-band spectrum rights	Nordic nRF9151 cellular IoT module	[93]
Iridium Communications	LEO	Coordinated L-band spectrum	NB-IoT and D2D devices	[86]
Deutsche Telekom	GEO/LEO	n249, n255 (L-band), n256 (S-band)	Nordic nRF9151	[94]
Mavenir & Terrestar	GEO	S-band (2–4 GHz); 40 MHz MSS spectrum	Sony Altair ALT1250 module	[95]
Airbus & OQ Technology	LEO	S-band (2–4 GHz)	Drone-mounted user terminals	[74]

**Table 10:** NTN NB-IoT reported performance values (see Table 9 for deployment specifics).

Performance Metric	Reported Value and Capability	Deplyment
Packet Delivery Ratio	90–95% in UAV BVLOS field trials	UAV Field Trial [102]
Coverage	∼70 million km^2^, 37 countries, 5 continents	Skylo [103]
	36 countries, >60 million km^2^	Skylo [92]
	100% global coverage	Iridium [86]
Device Capacity	Skylo GEO: ∼20 packets/min (up to 1200 bytes each)	Skylo [104]
	Max 12 devices can simultaneously send/receive over the carrier	Kyocera AVX [105]
Throughput	LEO (600–900 km): 20–40 kbps (comparable to terrestrial NB-IoT)	Skylo [92]
	5 kbps with 99.95% continuity (LEO, S-band drone demo)	OQ Technology [74]
Latency (GEO)	∼600 ms RTT	Skylo [103]
	5–10 s end-to-end	Skylo [104]
Latency (LEO)	<1 s for small messages (Iridium NTN Direct)	Iridium [86]
	Lower latency and higher data rates than GEO	Deutsche Telekom [89]
	Seconds in UAV field trials	UAV Field Trial [102]
Power Consumption	4–10 mAH per 10 satellite messages	Skylo [106]
	Multi-year battery life; comparable to terrestrial in poor coverage areas	Skylo [106]
	Hundreds of mW in UAV field trials	UAV Field Trial [102]
Signal Quality	Indoor not supported without specialized external antennas	Skylo [103]
	Works in adverse weather; line-of-sight preferred	Iridium [86]

## Data Availability

No new data were created or analyzed in this study. Data sharing is not applicable to this article.

## Abbreviations

The following abbreviations are used in this manuscript:

3GPP	3rd Generation Partnership Project
5GC	5G Core
AI	Artificial Intelligence
AMF	Access and Mobility Management Function
ARQ	Automatic Repeat Request
BER	Bit Error Rate
BLER	Block Error Rate
BVLOS	Beyond Visual Line of Sight
CapEx	Capital Expenditure
Cat M1	LTE Category M1 (LTE-M)
Cat NB	Narrowband IoT Category (NB1/NB2)
CB-EDT	Contention-Based Early Data Transmission
CFO	Carrier Frequency Offset
CNN	Convolutional Neural Network
CNR	Carrier-to-Noise Ratio
COTS	Commercial Off-The-Shelf
CP	Control Plane
CU	Central Unit (gNB-CU)
DL	Downlink
DM-RS	Demodulation Reference Signal
DRL	Deep Reinforcement Learning
DU	Distributed Unit (gNB-DU)
ECSS	European Cooperation for Space Standardization
ED	End Device
EDT	Early Data Transmission
eDRX	extended Discontinuous Reception
EIRP	Effective Isotropic Radiated Power
eMBB	enhanced Mobile Broadband
eMTC	enhanced Machine Type Communication
ESA	European Space Agency
EU	European Union
F1	Interface between gNB-CU and gNB-DU
FAA	Federal Aviation Administration
FCC	Federal Communications Commission
FD	Full Duplex
FDD	Frequency Division Duplex
FPGA	Field-Programmable Gate Array
FSPL	Free Space Path Loss
GEO	Geostationary Earth Orbit
gNB	Next-generation Node B (5G base station)
GNSS	Global Navigation Satellite System
GPS	Global Positioning System
G/T	Figure of Merit (Gain-to-Noise Temperature)
HAPS	High-Altitude Platform Station
HARQ	Hybrid Automatic Repeat Request
HD	Half Duplex
HFL	Hierarchical Federated Learning
HPUE	High-Power User Equipment
HTS	High-Throughput Satellite
IAB	Integrated Access and Backhaul
ICI	Inter-Carrier Interference
IoT	Internet of Things
IRIS^2^	Infrastructure for Resilience, Interconnectivity and Security by Satellite
ISL	Inter-Satellite Link
ITU	International Telecommunication Union
LEO	Low Earth Orbit
LMS	Land Mobile Satellite
LPWA	Low-Power Wide-Area
LTE-M	LTE for Machines
M2M	Machine-to-Machine
MAC	Medium Access Control
MANO	Management and Orchestration
MCL	Maximum Coupling Loss
MCS	Modulation and Coding Scheme
MEO	Medium Earth Orbit
MIL-HDBK	Military Handbook
MIMO	Multiple-Input Multiple-Output
mMTC	massive Machine-Type Communication
MTC	Machine-Type Communication
NASA	National Aeronautics and Space Administration
NB-IoT	Narrowband Internet of Things
NFV	Network Function Virtualization
NG	Interface between gNB and 5GC
NIDD	Non-IP Data Delivery
NOMA	Non-Orthogonal Multiple Access
NPRACH	Narrowband Physical Random Access Channel
NTN	Non-Terrestrial Network
OAI	OpenAirInterface
OFDM	Orthogonal Frequency Division Multiplexing
OTFS	Orthogonal Time Frequency Space
PAA	Phased Array Antenna
PDCP	Packet Data Convergence Protocol
PHY	Physical layer
PLS	Physical Layer Security
PLR	Packet Loss Rate
PNT	Positioning, Navigation, and Timing
PSM	Power-Saving Mode
PUSCH	Physical Uplink Shared Channel
QAM	Quadrature Amplitude Modulation
QPSK	Quadrature Phase Shift Keying
RACH	Random Access Channel
RAN	Radio Access Network
RAN3	3GPP Radio Access Network Working Group 3
RIS	Reconfigurable Intelligent Surface
RLC	Radio Link Control
RRC	Radio Resource Control
RSRP	Reference Signal Received Power
RTT	Round-Trip Time
RU	Resource Unit
SA1	3GPP Service and System Aspects Working Group 1
S&F	Store and Forward
SAM	Satellite-Assisted Manner
SCS	Subcarrier Spacing
SDM	Satellite-Dedicated Manner
SDN	Software-Defined Networking
SEE	Single Event Effects
SI	Study Item
SNR	Signal-to-Noise Ratio
SRI	Satellite Radio Interface
STO	Symbol Timing Offset
SWaP	Size, Weight, and Power
TDD	Time Division Duplex
TID	Total Ionizing Dose
TN	Terrestrial Network
TR	Technical Report
TS	Technical Specification
UAS	Unmanned Aerial Systems
UAV	Unmanned Aerial Vehicle
UE	User Equipment
UL	Uplink
UP	User Plane
uRLLC	ultra-reliable low-latency communication
VIAVI	VIAVI Solutions
VSAT	Very Small Aperture Terminal
WI	Work Item
Xn	Interface between gNBs
